# Oleanolic Acid Glycosides from *Scabiosa caucasica* and *Scabiosa ochroleuca*: Structural Analysis and Cytotoxicity

**DOI:** 10.3390/molecules28114329

**Published:** 2023-05-25

**Authors:** Samvel Nazaryan, Antoine Bruguière, Nelli Hovhannisyan, Tomofumi Miyamoto, Alexandre M. M. Dias, Pierre-Simon Bellaye, Bertrand Collin, Loïc Briand, Anne-Claire Mitaine-Offer

**Affiliations:** 1 Center for Taste and Feeding Behavior (CSGA), CNRS, INRAE, Agro Institute, Université de Bourgogne, 21000 Dijon, France; samvel.nazaryan@univ-fcomte.fr (S.N.); antoine.bruguiere@u-bourgogne.fr (A.B.); loic.briand@inrae.fr (L.B.); 2 Center of Excellence in Applied Biosciences, Yerevan State University (YSU), Yerevan 0025, Armenia; nellihovhannisyannh@gmail.com; 3 Graduate School of Pharmaceutical Sciences, Kyushu University, Fukuoka 819-0395, Japan; miyamoto@phar.kyushu-u.ac.jp; 4 Plateforme d’Imagerie et de Radiothérapie Précliniques, Service de Médecine Nucléaire, Centre Georges-François Leclerc, 21000 Dijon, France; aldias@cgfl.fr; 5 Plateforme d’Imagerie et de Radiothérapie Précliniques, Service de Médecine Nucléaire, Centre Georges-François Leclerc, UMR INSERM, Université de Bourgogne, Institut Agro 1231, 21000 Dijon, France; psbellaye@cgfl.fr; 6 Plateforme d’Imagerie et de Radiothérapie Précliniques, Service de Médecine Nucléaire, Centre Georges-François Leclerc, ICMUB, UMR CNRS, Université de Bourgogne 6302, 21000 Dijon, France; bertrand.collin@u-bourgogne.fr

**Keywords:** *Scabiosa caucasica*, *Scabiosa ochroleuca*, oleanolic acid, glycosides, cytotoxicity

## Abstract

In the field of research on medicinal plants from the Armenian flora, the phytochemical study of two *Scabiosa* L. species, *S. caucasica* M. Bieb. and *S. ochroleuca* L. (Caprifoliaceae), has led to the isolation of five previously undescribed oleanolic acid glycosides from an aqueous-ethanolic extract of the roots: 3-*O*-α-L-rhamnopyranosyl-(1→3)-β-D-glucopyranosyl-(1→4)-β-D-glucopyranosyl-(1→4)-β-D-xylopyranosyl-(1→3)-α-L-rhamnopyranosyl-(1→2)-α-L-arabinopyranosyloleanolic acid 28-*O*-β-D-glucopyranosyl-(1→6)-β-D-glucopyranosyl ester, 3-*O*-β-D-xylopyranosyl-(1→2)-[α-L-rhamnopyranosyl-(1→4)]-β-D-glucopyranosyl-(1→4)-β-D-glucopyranosyl-(1→4)-β-D-xylopyranosyl-(1→3)-α-L-rhamnopyranosyl-(1→2)-α-L-arabinopyranosyloleanolic acid 28-*O*-β-D-glucopyranosyl-(1→6)-β-D-glucopyranosyl ester, 3-*O*-β-D-xylopyranosyl-(1→2)-[α-L-rhamnopyranosyl-(1→4)]-β-D-glucopyranosyl-(1→4)-β-D-glucopyranosyl-(1→4)-β-D-xylopyranosyl-(1→3)-α-L-rhamnopyranosyl-(1→2)-α-L-arabinopyranosyloleanolic acid, 3-*O*-β-D-xylopyranosyl-(1→2)-[α-L-rhamnopyranosyl-(1→4)]-β-D-xylopyranosyl-(1→4)-β-D-glucopyranosyl-(1→4)-β-D-xylopyranosyl-(1→3)-α-L-rhamnopyranosyl-(1→2)-α-L-arabinopyranosyloleanolic acid 28-*O*-β-D-glucopyranosyl-(1→6)-β-D-glucopyranosyl ester, 3-*O*-α-L-rhamnopyranosyl-(1→4)-β-D-glucopyranosyl-(1→4)-β-D-glucopyranosyl-(1→4)-β-D-xylopyranosyl-(1→3)-α-L-rhamnopyranosyl-(1→2)-α-L-arabinopyranosyloleanolic acid 28-*O*-β-D-glucopyranosyl-(1→6)-β-D-glucopyranosyl ester. Their full structural elucidation required extensive 1D and 2D NMR experiments, as well as mass spectrometry analysis. For the biological activity of the bidesmosidic saponins and the monodesmosidic saponin, their cytotoxicity on a mouse colon cancer cell line (MC-38) was evaluated.

## 1. Introduction

As a developing country, Armenia still faces difficulty in combatting major diseases such as cancer, diabetes, and heart disease. According to the reports of the World Health Organization and the International Agency for Research on Cancer, the crude cancer incidence rate in 2020 was estimated to be 312.5 cases per 100,000 individuals in Armenia [1]. The types of cancer with the highest frequency among females were breast, colorectal, and cervical cancer, while among males, they were lung, bladder, and colorectal cancer [2]. The need for new anti-cancer agents is thus important, and one way to develop them is to search for cytotoxic compounds from natural sources. Saponins are one of the groups of secondary metabolites known and studied for their in vitro cytotoxicity [3,4,5,6]. To select plants rich in saponins, we turned to those used in traditional Armenian medicine. Most of the traditional medicines’ uses are based on the oral tradition, but also have many practical applications and are supported by data from the scientific literature. Armenian folk medicine contains a vast amount of information on the medicinal properties of local plants. Among them, the genus *Scabiosa* L.—especially *S. caucasica* M. Bieb—is one of the herbs most recognized in traditional Armenian medicine for its ability to heal skin irritation, scabies, and the symptoms of influenza [7]. The *Scabiosa* genus is included in the Caprifoliaceae family, of the Dipsacales order, and consists of approximately 80 annual or perennial species, distributed in Europe, Asia, and Eastern and Southern Africa [8]. *S. caucasica* (Caucasian pincushion or Armenian Chrysanthemum) and *S. ochroleuca* L. (White-yellow pincushion), are perennial species. They are wild *Scabiosa*, growing in high mountain meadows, at elevations of over 2500 m, in the Caucasus and Transcaucasus, but one can also find them in greenhouses and floral markets. The name, *Scabiosa caucasica* M. Bieb, was used for *Lomelosia caucasica* (M. Bieb.) Greuter & Burdet, for many years. Following an examination of the Dipsacaceae family, some species from *Scabiosa* were reclassified, such as *S. caucasica*, which was instead placed in the *Lomelosia* Raf. genus [9]. To maintain the connection between the report and the literature data, the synonym “*S. caucasica*” will be used in this article. From a phytochemical point of view, the *Scabiosa* genus is rich in saponins, and mono- and bidesmosidic triterpene glycosides have been previously isolated from *S. semipapposa* [10], *S. stellata* [11], *S. tschiliensis* [12], and *S. rotata* [13]. In order to complete the study of the saponins from the *Scabiosa* species, and to determine structure–activity relationships, a phytochemical study of *S. caucasica* and *S. ochroleuca* was carried out, and the cytotoxicity of the isolated compounds was evaluated on a mouse colon cancer cell line (MC-38).

## 2. Results and Discussion

**Figure 1:**
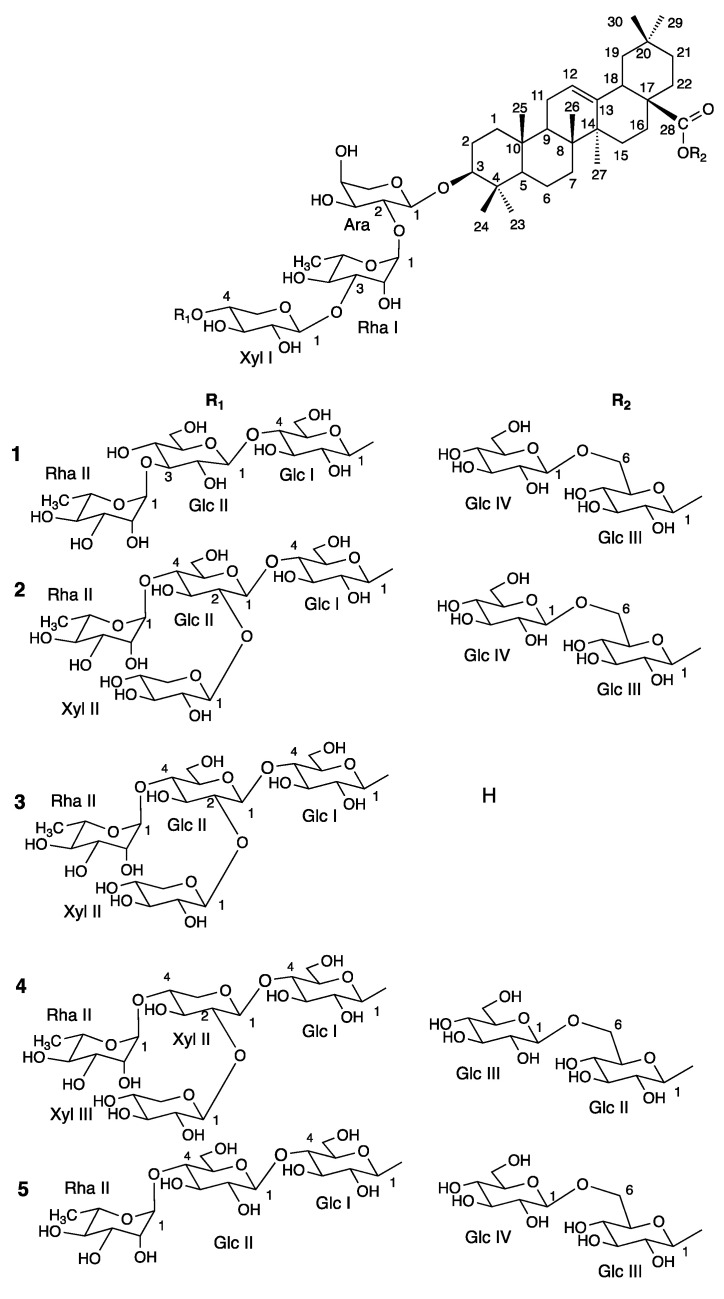
Structures of compounds **1**–**5**.

**Figure 2:**
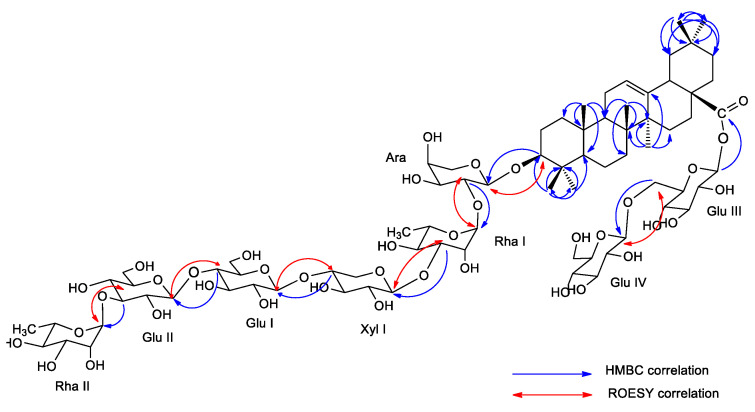
Key HMBC and ROESY correlations of compound **1**.

**Figure 3:**
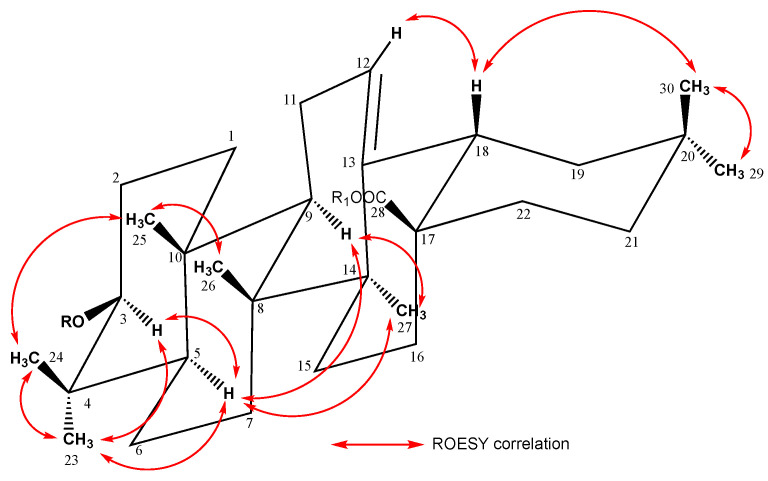
Key ROESY correlations for the aglycon of compound **1**.

Aqueous-ethanolic (35:75) extracts of the roots of *S. caucasica* and *S. ochroleuca* were purified by several chromatographic methods, allowing the isolation of five new glycosides. Their structures were established by 1D and 2D NMR analysis (^1^H, ^13^C, COSY, TOCSY, ROESY, HSQC, HMBC, see Supplementary Materials), in combination with mass spectrometry (ESI-MS). From *S. caucasica*, three bidesmosidic oleanolic acid glycosides (**1**, **4**, **5**) were obtained, and one mono- (**3**) and three bidesmosidic glycosides (**1**, **2**, **5**) were isolated from *S. ochroleuca*. For all the isolated compounds, **1**–**5** (Figure 1), the ^1^H- and ^13^C-NMR signals of the aglycone, assigned from the 2D NMR spectra, were in good agreement with those reported for the oleanolic acid (Figure 1, Figure 2 and Figure 3). For example, in compound **1**, the HSQC spectrum showed characteristic signals of seven angular methyl groups of a triterpene skeleton at *δ*_C_/*δ*_H_ 27.3/1.05 (s) (CH_3_-23), 15.8/0.88 (s) (CH_3_-24), 14.8/0.99 (s) (CH_3_-25), 16.6/0.83 (s) (CH_3_-26), 24.9/1.18 (s) (CH_3_-27), 32.1/0.93 (s) (CH_3_-29), 22.7/0.96 (s) (CH_3_-30), and signals of an ethylene bond at *δ*_C_/*δ*_H_ 122.4/5.27 (br t, *J* = 3.6 Hz) (CH-12). The signal of an ester function at the C-28 position at *δ*_C_ 178.0 ppm, and the chemical shift of the C-3 at *δ*_C_ 89.2 ppm, due to a *O*-heterosidic linkage, suggested that compound **1** was a bidesmosidic glycoside of oleanolic acid (Table 1). The configurations of C-3, C-5 C-9, and C-18 were determined using the ROE correlations with the angular methyl groups (Figure 3), such as the cross-peaks between the H-3 α-axial and the H_3_-23 α-equatorial, the H-5 α-axial and the H_3_-23 α-equatorial, the H-9 α-axial and the H_3_-27 α-axial, and the H-18 β-axial and the H_3_-30 β-axial. The α or β configurations of the other protons of the aglycon were also determined with the ROESY spectrum (Table 1).

For compounds **1**–**5**, each monosaccharide, linked at the 3 and 28 positions of the oleanolic acid, was identified by extensive 2D NMR analysis, mainly COSY and TOCSY experiments, as α-L-arabinopyranosyl (Ara), α-L-rhamnopyranosyl (Rha), β-D-xylopyranosyl (Xyl), and β-D-glucopyranosyl (Glc) (Table 2). The relatively large ^3^*J*_H-1, H-2_ values of the Glc, Xyl, and Ara (5.0–8.2 Hz) indicated a β anomeric orientation for Glc and Xyl, and an α anomeric orientation for Ara. The large ^1^*J*_H-1,C-1_ values of the Rha (165–168 Hz), confirmed that the anomeric protons were equatorial (α-pyranoid anomeric form). The absolute configurations of the sugars were determined to be D for Xyl and Glc, and L for Ara and Rha (Experimental section).

The complete structural analysis is detailed below for the five glycosides isolated from *S. caucasica* and *S. ochroleuca*.

The mass spectrum of compound **1** in ESIMS (positive mode) revealed a quasi-molecular ion peak at *m*/*z* 1683.7553 [M + Na]^+^, in agreement with the molecular formula, C_76_H_124_O_39_. This suggests a molecular mass of 1660.

The glycosidic part of compound **1** is composed of eight residues, as revealed by the HSQC correlations at δ_H_/δ_C_ 5.38 (d, *J* = 8.1 Hz)/94.4, 5.21 (s)/100.1, 5.19 (s)/101.4, 4.55 (d, *J* = 7.6 Hz)/104.9, 4.52 (d, *J* = 5.0 Hz)/103.8, 4.43 (d, *J* = 7.8 Hz)/103.0, 4.42 (d, *J* = 7.7 Hz)/101.7, and 4.37 (d, *J* = 7.7 Hz)/103.2 ppm. The first sugar unit, linked at the C-3 position of the aglycon, was identified as an Ara, based on the HMBC cross-peaks at δ_H_/δ_C_ 4.52 (Ara H-1)/89.2 (C-3) and 3.14 (dd, *J* = 11.5, 4.0 Hz) (H-3)/103.8 (Ara C-1). This was proven by the ROE correlation at δ_H_/δ_H_ 4.52 (Ara H-1)/3.14 (H-3). To determine the oligosaccharidic chain linked to this Ara, our observations were based on the correlations observed in the HMBC and ROESY spectra. The HMBC spectrum of compound **1** displayed correlations at δ_H_/δ_C_ 5.21 (Rha I H-1)/74.9 (Ara C-2), and reverse correlations at 3.79 (Ara H-2)/100.1 (Rha I C-1), 4.55 (Xyl I H-1)/80.7 (Rha I C-3), 4.42 (Glc I H-1)/76.9 (Xyl I C-4), 4.43 (Glc II H-1)/78.9 (Glc I C-4), and 5.19 (Rha II H-1)/82.7 (Glc II C-3). The structure of this chain was verified by ROESY cross-peaks at δ_H_/δ_H_ at 5.21 (Rha I H-1)/3.79 (Ara H-2), 4.55 (Xyl I H-1)/3.87 (Rha I H-3), 4.42 (Glc I H-1)/3.72 (Xyl I H-4), 4.43 (Glc II H-1)/3.58 (Glc I H-4), 5.19 (Rha II H-1)/3.53 (Glc II H-3). For the glycosidic part, linked at the ester function at C-28, the HMBC cross-peak at δ_H_/δ_C_ 5.38 (Glc III H-1)/178.0 (C-28), revealed the presence of a β-D-glucopyranosyl unit. The deshielded value of the position C-6 of this sugar at 68.1, and the HMBC cross-peak at δ_H_/δ_C_ 4.37 (Glc IV H-1)/68.1 (Glc III C-6), suggested the 28-*O*-β-D-glucopyranosyl-(1→6)-β-D-glucopyranosyl sequence. This was confirmed by the NOE correlation at δ_H_/δ_H_ 4.37 (Glc IV H-1)/3.79, 4.15 (Glc III H_2_-6). According to this analysis, the structure of compound **1** is established as 3-*O*-α-L-rhamnopyranosyl-(1→3)-β-D-glucopyranosyl-(1→4)-β-D-glucopyranosyl-(1→4)-β-D-xylopyranosyl-(1→3)-α-L-rhamnopyranosyl-(1→2)-α-L-arabinopyranosyloleanolic acid 28-*O*-β-D-glucopyranosyl-(1→6)-β-D-glucopyranosyl ester (Figure 1 and Figure 2).

The mass spectrum of compound **2** in ESIMS (positive mode) showed a quasi-molecular ion peak at *m*/*z* 1815.8003 [M + Na]^+^, suggesting a molecular formula of C_81_H_132_O_43_, and a molecular mass of 1792. The difference of 132 u between **1** and **2** is related to a supplementary pentose moiety in the oligosaccharidic chain. The assignments of all the NMR signals of the molecule **2** revealed a structure almost superimposable to **1**, except for the Glc II. In fact, the chemical shifts of the positions 2 and 4 were deshielded at δ_C_/δ_H_ 76.7/3.44 and 76.9/3.74, respectively. HMBC correlations between δ_H_/δ_C_ 4.38 (d, *J* = 7.8 Hz) (Xyl II H-1) and 76.7 (Glc II C-2), and between δ_H_/δ_C_ 5.37 (s) (Rha II H-1) and 76.9 (Glc II C-4) proved the 2,4 disubstitution of the Glc II. Therefore, the structure of **2** is established as 3-*O*-β-D-xylopyranosyl-(1→2)-[α-L-rhamnopyranosyl-(1→4)]-β-D-glucopyranosyl-(1→4)-β-D-glucopyranosyl-(1→4)-β-D-xylopyranosyl-(1→3)-α-L-rhamnopyranosyl-(1→2)-α-L-arabinopyranosyloleanolic acid 28-*O*-β-D-glucopyranosyl-(1→6)-β-D-glucopyranosyl ester.

For compound **3**, a quasi-molecular ion peak at *m*/*z* 1491.6949 [M + Na]^+^ in ESIMS (positive mode) revealed a molecular formula of C_69_H_112_O_33_ and a molecular mass of 1468. The main difference with **2**, in the NMR spectra, is the value of δ_C_ 180.2 ppm of the carbonyl function at C28, which suggests a carboxylic group instead of an ester group in compound **2**. This was confirmed by the lack of NMR signals corresponding to the gentiobiose unit, 28-*O*-β-D-glucopyranosyl-(1→6)-β-D-glucopyranosyl ester, which led to the conclusion of a monodesmosidic saponin, 3-*O*-β-D-xylopyranosyl-(1→2)-[α-L-rhamnopyranosyl-(1→4)]-β-D-glucopyranosyl-(1→4)-β-D-glucopyranosyl-(1→4)-β-D-xylopyranosyl-(1→3)-α-L-rhamnopyranosyl-(1→2)-α-L-arabinopyranosyloleanolic acid.

The mass spectrum of compound **4** in ESIMS (positive mode) showed a quasi-molecular ion peak at *m*/*z* 1785.7940 [M + Na]^+^, suggesting a molecular formula of C_80_H_130_O_42_, and a molecular mass of 1762. The comparison of the molecular weight of compounds **2** and **4**, showed a difference of 30 u, which suggests the presence of a pentosyl group in **4**, instead of a hexosyl group in **2**. For the structure, the NMR signals of the main part of the molecule corresponding to 3-*O*-β-D-glucopyranosyl-(1→4)-β-D-xylopyranosyl-(1→3)-α-L-rhamnopyranosyl-(1→2)-α-L-arabinopyranosyloleanolic acid 28-*O*-β-D-glucopyranosyl-(1→6)-β-D-glucopyranosyl ester, are almost superimposable. The difference is located at the last three sugars of the 3-*O*-heterosidic chain. For compound **4**, two β-D-xylopyranosyl (Xyl II and Xyl III), and one α-L-rhamnopyranosyl (Rha II) moieties were found, identified mainly by TOCSY, COSY, and HSQC spectra. Xyl II is linked to the Glc I moiety at the 4 position, according to the HMBC correlation between δ_H_/δ_C_ 4.34 (d, *J* = 7.6 Hz) (Xyl II H-1) and 78.8 (Glc I C-4). Moreover, cross-peaks at δ_H_/δ_C_ 4.25 (d, *J* = 7.6 Hz) (Xyl III H-1)/77.0 (Xyl II C-2), and δ_H_/δ_C_ 3.62 (Xyl II H-4)/100.9 (Rha II C-1), were allowed to establish the β-D-xylopyranosyl-(1→2)-[α-L-rhamnopyranosyl-(1→4)]-β-D-xylopyranosyl chain. Based on these conclusions, compound **4** is identified as 3-*O*-β-D-xylopyranosyl-(1→2)-[α-L-rhamnopyranosyl-(1→4)]-β-D-xylopyranosyl-(1→4)-β-D-glucopyranosyl-(1→4)-β-D-xylopyranosyl-(1→3)-α-L-rhamnopyranosyl-(1→2)-α-L-arabinopyranosyloleanolic acid 28-*O*-β-D-glucopyranosyl-(1→6)-β-D-glucopyranosyl ester.

In the case of compound **5**, this molecule shares the same molecular mass of 1660 as compound **1**, corresponding to C_76_H_124_O_39_. When the total assignment of the protons and carbons of compound **5** was achieved by 2D NMR, the only difference was located at the Glc II moiety. A deshielded value of δ_C_ 77.0 for Glc II-4, and an HMBC cross-peak at δ_H_/δ_C_ 5.40 (s) (Rha II-1)/77.0 (Glc II-4), suggested this specific linkage. Thus, the structure of **5** was established as 3-*O*-α-L-rhamnopyranosyl-(1→4)-β-D-glucopyranosyl-(1→4)-β-D-glucopyranosyl-(1→4)-β-D-xylopyranosyl-(1→3)-α-L-rhamnopyranosyl-(1→2)-α-L-arabinopyranosyloleanolic acid 28-*O*-β-D-glucopyranosyl-(1→6)-β-D-glucopyranosyl ester.

The saponins **1**–**5**, found in *S. caucasica* and *S. ochroleuca*, have structural similarities with those found in *S. semipapposa* [10], *S. stellata* [11], and *S. tschiliensis* [12]. They share the same aglycone, an oleanolic acid with a gentiobiose unit attached to the C28 of oleanolic acid for **1**, **2**, **4**, **5**, and also share similar sets of the first three osidic units in the oligosaccharide chains attached to the C3 position, such as the sequence 3-*O*-β-D-xylopyranosyl-(1→3)-α-L-rhamnopyranosyl-(1→2)-α-L-arabinopyranosyloleanolic acid 28-*O*-β-D-glucopyranosyl-(1→6)-β-D-glucopyranosyl ester. Surprisingly, these structures are close to those isolated from *Weigela* species, such as *W. stelzneri* and *W.* × “Bristol Ruby” [4,5]. The *Scabiosa* and *Weigela* genera belong to the same Caprifoliaceae family of the Dipsacales order, but their subfamily is different—Dipsacoideae for *Scabiosa* and Diervilloideae for *Weigela*. From a chemotaxonomic point of view, we must continue our investigations to determine whether this type of sequence could represent a chemotaxonomic marker of these two subfamilies.

**Figure 4:**
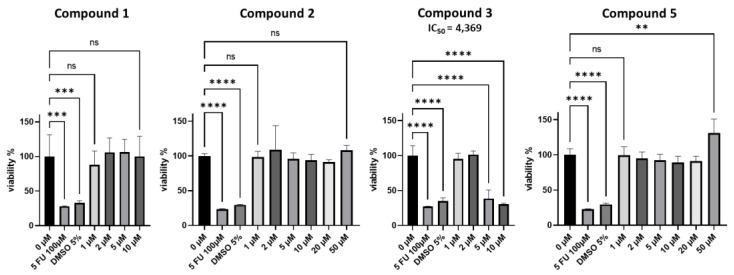
Evaluation of the cytotoxicity on a mouse colon cancer cell line (MC-38). Statistical analyses were performed using a one-way ANOVA test in GraphPad Prism 9 software (San Diego, CA, USA). Statistically significant results are annotated, with ** *p* < 0.01 *** *p* < 0.001 and **** *p* < 0.0001; and ns—not significant. 5-FU: 5-FluoroUracile. DMSO: DiMethylSulfOxyde.

The cytotoxicity of the bidesmosidic saponins, **1**, **2**, **5**, and the monodesmosidic, **3**, was evaluated on a mouse colon cancer cell line (MC-38). Only compound **3** showed potent cytotoxicity, with an IC_50_ of 4.37 µM, compared to 5-FU with an IC_50_ of 11.7 µM. These results highlight the key role of the lack or presence of an esterification of the C-28 position (Figure 4). The cytotoxicity of saponins is often related to their ability to interact with membranes. This interaction seems to usually be lowered when the saponin possess a second sugar chain [6]. Among the tested molecules, compound **3** is the only monodesmosidic saponin. This would explain why compound **3** is active, compared with the bidesmosidic **2**, which has the same structure but, with an additional sugar chain at the C-28 position. The other structural differences between the compounds are the number of sugars linked to the genin at the C3 position (6 or 7 sugars), and the nature and the linkage of the last three sugars of the oligosaccharidic chain. Those structural elements do not seem to influence their activity, as none of them are cytotoxic.

## 3. Materials and Methods

### 3.1. General Experimental Procedures

Optical rotation values were recorded on an AA-10R automatic polarimeter (Optical Activity LTD, Huntingdon, UK). Melting points were measured with a Banc Kofler (Wagner and Muntz, München, Germany). NMR spectra were recorded on a Varian INOVA 600 MHz spectrometer (Agilent Technologies, Santa Clara, CA, USA) equipped with 3 mm triple resonance inverse and 3 mm dual broadband probe heads. Spectra were recorded in methanol-*d*_4_, and all spectra were recorded at T = 308.15 K. Pulse sequences were taken from the Varian pulse sequence library (gCOSY, gHSQCAD, and gHMBCAD with adiabatic pulses, CRISIS-HSQC and CRISIS-HMBC). TOCSY spectra were acquired using a DIPSI spin-lock and 150 ms mixing time. Mixing time in ROESY experiments was 300 ms. Chemical shifts were reported in δ units and coupling constants (*J*) in Hz. ESIMS (positive-ion mode) was performed on a ThermoScientific (Waltham, MA, USA)—Orbitrap Fusion apparatus. Isolations of the compounds were carried out using column chromatography (CC) with Sephadex LH-20 (550 mm × 20 mm, GE Healthcare Bio-Sciences AB, Uppsala, Sweden), and vacuum liquid chromatography (VLC) with reversed-phase RP-18 silica gel (75–200 μm, Silicycle, Quebec City, QC, Canada). Medium-pressure liquid chromatography (MPLC) was performed using silica gel 60 (Merck, Darmstadt, Germany, 15–40 μm) with a Gilson M 305 pump (25 SC head pump, M 805 manometric module), a Büchi glass column (460 mm × 25 mm and 460 mm × 15 mm), and a Büchi precolumn (110 mm × 15 mm). Thin-layer chromatography (TLC, Silicycle) and high-performance thin-layer chromatography (HPTLC, Merck) were carried out on precoated silica gel plates (60 F_254_, solvent system CHCl_3_/MeOH/H_2_O (64:40:8)). The spray reagent for saponins was vanillin reagent (1% vanillin in EtOH/H_2_SO_4_, 50:1). The HPLC was performed on an Agilent 1260 instrument, equipped with a degasser, a quaternary pump, a sample changer, and a UV detector (210 nm). The chromatographic separation for the analytical part was carried out on a C18 column (250 mm × 4.6 mm internal diameter, 5 μm; Phenomenex LUNA, Torrance, CA, USA) at room temperature and protected by a guard column. The mobile phase consists of (A) 0.01% (*v*/*v*) aqueous trifluoroacetic acid, and (B) acetonitrile delivered at 1 mL/min, going from 30% to 80% B in 30 min. The injection volume was 10 µL.

### 3.2. Plant Material

*Scabiosa caucasica* and *S. ochroleuca* were purchased from Pépinière Lepage**^®^** (Les Ponts-de-Cé, France) in April 2021, and samples were deposited in the herbarium at the Laboratory of Pharmacognosy, Université de Bourgogne Franche-Comté, Dijon, France, under the numbers No. 2021/04/09 and 2021/04/10, respectively.

### 3.3. Extraction and Isolation

The aerial parts of *S. caucasica* (SC) and *S. ochroleuca* (SO) were separated from the roots. The leaves and roots were dried and ground into a fine powder. A previous screening, by TLC, of an aqueous ethanolic extract of the leaves, showed the lack of saponins in them. Four successive macerations at room temperature (EtOH/H_2_O-75:35) were performed with the roots of (SC) (25.81 g powder, 200 mL solvent) and (SO) (144.10 g powder, 550 mL solvent), yielding 5.89 g and 8.04 g of extracts, respectively. The same chromatographic protocol was applied for each extract. A total of 3.00 g of the (SC) extract and 5.00 g of the (SO) extract were separated using RP-18 silica gel with a H_2_O/EtOH gradient (100:0, 75:25, 50:50, 25:75, 0:100). According to their TLC profiles, fractions were combined, leading to SC1 (454.0 mg); SC2 (553.7 mg); SC3 (114.4 mg); and SC4 (75.6 mg) for (SC), and SO1 (385.2 mg); SO2 (1392.2 mg); and SO3 (741.0 mg) for (SO). Fractions, SC2 and SO2, were further purified by CC, with Sephadex LH-20, using ethanol. SC2.1 (2.8 mg); SC2.2 (5.8 mg); SC2.3 (8.0 mg); SC2.4 (9.7 mg); SC2.5 (147.5 mg); SC2.6 (180.6 mg); and SC2.7 (11.3 mg) for (SC), and SO2.1 (120.5 mg); SO2.2 (544.3 mg); SO2.3 (386.3 mg); and SO2.4 (102.2 mg) for (SO) were thus obtained. The final purification steps were performed on fractions, SC2.5, SC2.6, SO2.1, and SO2.2, using successive MPLCs with silica gel 60 (CHCl_3_/MeOH/H_2_O 64:40:8, flow 2.5 mL/min), yielding compounds, 1 (6.8 mg); 4 (4.0 mg); and 5 (7.0 mg) from (SC), and 1 (13.2 mg); 2 (47.9 mg); 3 (19.6 mg); and 5 (6.2 mg) from (SO). The purity of each of the saponins was estimated using HPTLC and HPLC.

*3-O-α-L-rhamnopyranosyl-(1→3)-β-D-glucopyranosyl-(1→4)-β-D-glucopyranosyl-(1→4)-β-D-xylopyranosyl-(1→3)-α-L-rhamnopyranosyl-(1→2)-α-L-arabinopyranosyloleanolic acid 28-O-β-D-glucopyranosyl-(1→6)-β-D-glucopyranosyl ester* (**1**). White, amorphous powder. Melting point, 220°C. [α]^25^_D_-15 (c 0.2, MeOH). For ^1^H and ^13^C NMR data (600 MHz, CD_3_OD), see Table 1 and Table 2. ESIMS (positive-ion mode) *m*/*z*: 1683.7553 [M + Na]^+^ (calcd. for C_76_H_124_O_39_Na, 1683,7617).

*3-O-β-D-xylopyranosyl-(1→2)-[α-L-rhamnopyranosyl-(1→4)]-β-D-glucopyranosyl-(1→4)-β-D-glucopyranosyl-(1→4)-β-D-xylopyranosyl-(1→3)-α-L-rhamnopyranosyl-(1→2)-α-L-arabinopyranosyloleanolic acid 28-O-β-D-glucopyranosyl-(1→6)-β-D-glucopyranosyl ester* (**2**). White, amorphous powder. Melting point, 238°C. [α]^25^_D_-10 (c 0.2, MeOH). For ^1^H and ^13^C NMR data (600 MHz, CD_3_OD), see Table 1 and Table 2. ESIMS (positive-ion mode) *m*/*z*: 1815.8003 [M + Na]^+^ (calcd. for C_81_H_132_O_43_Na, 1815.8040).

*3-O-β-D-xylopyranosyl-(1→2)-[α-L-rhamnopyranosyl-(1→4)]-β-D-glucopyranosyl-(1→4)-β-D-glucopyranosyl-(1→4)-β-D-xylopyranosyl-(1→3)-α-L-rhamnopyranosyl-(1→2)-α-L-arabinopyranosyloleanolic acid* (**3**). White, amorphous powder. Melting point, 250 °C. [α]^25^_D_-17 (c 0.2, MeOH). For ^1^H and ^13^C NMR data (600 MHz, CD_3_OD), see Table 1 and Table 2. ESIMS (positive-ion mode) *m*/*z*: 1491.6949 [M + Na]^+^ (calcd. for C_69_H_112_O_33_Na, 1491.6984).

*3-O-β-D-xylopyranosyl-(1→2)-[α-L-rhamnopyranosyl-(1→4)]-β-D-xylopyranosyl-(1→4)-β-D-glucopyranosyl-(1→4)-β-D-xylopyranosyl-(1→3)-α-L-rhamnopyranosyl-(1→2)-α-L-arabinopyranosyloleanolic acid 28-O-β-D-glucopyranosyl-(1→6)-β-D-glucopyranosyl ester* (**4**). White, amorphous powder. [α]^25^_D_-20 (c 0.2, MeOH). For ^1^H and ^13^C NMR data (600 MHz, CD_3_OD), see Table 1 and Table 2. ESIMS (positive-ion mode) *m*/*z*: 1785.7940 [M + Na]^+^ (calcd. for C_80_H_130_O_42_Na, 1785.7934).

*3-O-α-L-rhamnopyranosyl-(1→4)-β-D-glucopyranosyl-(1→4)-β-D-glucopyranosyl-(1→4)-β-D-xylopyranosyl-(1→3)-α-L-rhamnopyranosyl-(1→2)-α-L-arabinopyranosyloleanolic acid 28-O-β-D-glucopyranosyl-(1→6)-β-D-glucopyranosyl ester* (**5**). White, amorphous powder. Melting point, 226°C. [α]^25^_D_-12 (c 0.2, MeOH). For ^1^H and ^13^C NMR data (600 MHz, CD_3_OD), see Table 1 and Table 2. ESIMS (positive-ion mode) *m*/*z*: 1683.7553 [M + Na]^+^ (calcd. for C_76_H_124_O_39_Na, 1683,7617).

### 3.4. Acid Hydrolysis and Absolute Configuration Determination

An aliquot (150 mg) of a fraction-rich saponin was hydrolyzed with 2N aqueous CF_3_COOH (25 mL) for 3 h at 95 °C. After extraction with CH_2_Cl_2_ (3 × 15 mL), the aqueous layer was evaporated to dryness with H_2_O until neutral, to produce the sugar residue (40 mg). Arabinose, glucose, xylose, and rhamnose were identified by comparison with authentic samples by TLC using CH_3_COOEt/CH_3_COOH/CH_3_OH/H_2_O (65:25:15:15). After purification of these sugars, by prep-TLC in the same solvent, the optical rotation of each purified sugar was measured as follows: L-arabinose: *R*_f_ = 0.54, [α]^25^_D_ + 170 (*c* 0.2, H_2_O), D-glucose: *R*_f_ = 0.50, [α]^25^_D_ + 110 (*c* 0.2, H_2_O), D-xylose: *R*_f_ = 0.60, [α]^25^_D_ + 85 (*c* 0.2, H_2_O), and L-rhamnose: *R*_f_ = 0.69, [α]^25^_D_ + 10 (*c* 0.2, H_2_O).

### 3.5. Bioactivity Assay

MC-38 cells (mouse colon cancer, kerafast^®^ ENH204-FP) were seeded at the density of 2 × 10^4^ cells in 96 well plates (Falcon, Merck), and cultivated for 24 h before treatments in an RPMI 1640 medium (Corning, Corning, NY, USA) with 10% FBS (Dutcher, Limassol, Cyprus). Cells were then treated with the isolated compounds at the concentrations from 1 to 50 µM, for 48 h in an RPMI 1640 medium without FBS (200 µL per well). Viability was measured using an MTS colorimetric assay (Abcam, Waltham, MA, USA), following the manufacturer’s recommendations. Briefly, 20 µL of MTS reagent was added into each well, and optical density at 490 nm was read using a microplate reader (Spark^®^, Männedorf, Switzerland, Tecan), after 1 h incubation at 37 °C, 5% CO_2_, in the dark. The results were expressed as concentrations of compounds producing 50% toxicity (IC_50_ value). Fluorouracil (5-FU) was used as positive control and exhibited IC_50_ values of 11.7 µM. The experiment was conducted in quadruplicate.

## 4. Conclusions

The saponins, **1**–**5**, found in *S. caucasica* and *S. ochroleuca*, possess an oleanolic acid as aglycon, a gentiobiose unit attached to C28 for **1**, **2**, **4**, **5**, and the same first three osidic units in the oligosaccharide chain attached to C3, such as the sequence, 3-*O*-β-D-xylopyranosyl-(1→3)-α-L-rhamnopyranosyl-(1→2)-α-L-arabinopyranosyloleanolic acid 28-*O*-β-D-glucopyranosyl-(1→6)-β-D-glucopyranosyl ester. These glycosides have structural similarities with those found not only in other *Scabiosa* species from the Dipsacoideae subfamily of the Caprifoliaceae, but also with *Weigela* species, from the Diervilloideae. From a chemotaxonomic point of view, we need to continue our investigations to determine whether this type of sequence could represent a chemotaxonomic marker of these two subfamilies. Compound **3** exhibits potent cytotoxicity on a mouse colon cancer cell line (MC-38), with an IC_50_ of 4.37 µM, highlighting the key role of the lack or the presence of an esterification of the C-28 position.

## Figures and Tables

**Table 1:** ^13^C- and ^1^H-NMR data of the aglycon part in compounds **1**–**5** in CD_3_OD *.

Position	1		2		3		4		5	
	*δ*_C_	*δ*_H_	*δ*_C_	*δ*_H_	*δ*_C_	*δ*_H_	*δ*_C_	*δ*_H_	*δ*_C_	*δ*_H_
1(CH_2_)	38.5	α 1.01 m β 1.64 m	38.6	α 1.01 m β 1.64 m	38.5	α 0.98 m β 1.63 m	38.9	α 0.98 m β 1.62 m	38.5	α 0.98 m β 1.63 m
2(CH_2_)	25.8	β 1.75 m α 1.86 m	25.4	β 1.75 m α 1.87 m	22.4	β 1.72 m α 1.85 m	25.8	β 1.70 m α 1.83 m	26.1	β 1.70 m α 1.82 m
3(CH)	89.2	α 3.14 dd (11.5, 4.0)	89.2	α 3.14	89.2	α 3.13	89.2	α 3.11 dd (11.7, 4.1)	91.0	α 3.11 dd (11.8, 4.0)
4(C)	39.0	-	38.9	-	38.8	-	38.7	-	38.7	-
5(CH)	55.8	α 0.80	55.8	α 0.80	55.7	α 0.80 br d (11.2)	55.8	α 0.77	55.8	α 0.77
6(CH_2_)	18.0	β 1.42 α 1.56	18.1	β 1.43 α 1.56	18.2	β 1.43 α 1.56	18.0	β 1.42 α 1.54	18.0	β 1.49 α 1.54
7(CH_2_)	32.4	β 1.34 m α 1.49 m	32.6	β 1.35 m α 1.50 m	32.7	β 1.33 m α 1.53 m	32.5	β 1.32 m α 1.48 m	32.7	β 1.32 m α 1.48 m
8(C)	39.5	-	39.4	-	39.2	-	39.4	-	39.4	-
9(CH)	47.8	α 1.60	47.9	α 1.61	47.8	α 1.60	47.8	α 1.58	47.8	α 1.58
10(C)	36.7	-	36.6	-	36.5	-	36.5	-		-
11(CH_2_)	23.2	1.92 1.95	23.1	1.90 1.92	23.1	1.86 1.91	23.1	1.88 1.90	23.1	1.88 1.90
12(CH)	122.4	5.27 t (3.6)	122.4	5.28 t (3.6)	122.0	5.25 t (3.6)	122.4	5.24 t (3.4)	122.4	5.24 t (3.5)
13(C)	143.5	-	143.5	-	144.2	-	143.5	-	143.5	-
14(C)	41.4	-	41.5	-	41.6	-	41.5	-	41.4	-
15(CH_2_)	27.6	α 1.09 m β 1.81 m	27.5	α 1.10 m β 1.81 m	27.5	α 1.05 m β 1.79 m	27.6	α 1.08 m β 1.78 m	27.6	α 1.08 m β 1.78 m
16(CH_2_)	22.7	β 1.73 α 2.07 m	22.7	β 1.74 α 2.07 m	22.9	β 1.60 α 1.97 m	22.6	β 1.72 α 2.04 m	22.6	β 1.70 α 2.04 m
17(C)	46.7	-	46.7	-		-	46.5	-	46.4	-
18(CH)	41.2	β 2.89 dd (14.5, 3.3)	41.2	β 2.89 dd (12.7, 3.5)	41.5	β 2.87 dd (12.8, 3.4)	41.2	β 2.85 dd (13.3, 3.4)	41.2	β 2.85 dd (12.7, 3.3)
19(CH_2_)	45.9	β 1.16 α 1.74	45.9	β 1.18 α 1.74	46.2	β 1.13 α 1.68	45.8	β 1.14 α 1.71	45.8	β 1.13 α 1.70
20(C)	30.2	-	30.2	-	30.2	-	30.2	-	30.2	-
21(CH_2_)	33.6	β 1.24 α 1.42 m	33.5	β 1.25 α 1.43 m	33.7	β 1.20 α 1.40 m	33.5	β 1.23 α 1.40 m	33.8	β 1.21 α 1.39 m
22(CH_2_)	31.8	α 1.63 β 1.76	31.8	α 1.61 β 1.75	32.6	α 1.53 β 1.74	32.0	α 1.60 β 1.72	32.0	α 1.60 β 1.73
23(CH_3_)	27.3	α 1.05 s	27.3	α 1.05 s	27.3	α 1.05 s	27.1	α 1.02 s	27.1	α 1.02 s
24(CH_3_)	15.8	β 0.88 s	15.8	β 0.88 s	15.8	β 0.87 s	15.8	β 0.84 s	15.8	β 0.85 s
25(CH_3_)	14.8	β 0.99 s	14.8	β 0.99 s	14.7	β 0.97 s	14.9	β 0.96 s	14.8	β 0.96 s
26(CH_3_)	16.6	β 0.83 s	16.4	β 0.83 s	16.6	β 0.85 s	16.6	β 0.80 s	16.7	β 0.80 s
27(CH_3_)	24.9	α 1.18 s	24.9	α 1.18 s	25.1	α 1.18 s	24.9	α 1.15 s	24.9	α 1.15 s
28(CH_3_)	178.0	-	176.7	-	180.2	-	176.7	-	176.6	-
29(CH_3_)	32.1	α 0.93 s	32.1	α 0.94 s	32.2	α 0.92 s	32.0	α 0.90 s	32.0	α 0.90 s
30(CH_3_)	22.7	β 0.96 s	22.7	β 0.97 s	22.7	β 0.96 s	22.8	β 0.93 s	22.7	β 0.93 s

* Overlapped signals are reported without designated multiplicity. *δ* in ppm; *J* in parentheses in Hz.

**Table 2:** ^13^C- and ^1^H-NMR data of the osidic part, in compounds **1**–**5** in CD_3_OD *.

Position	1		2		3		4		5	
	*δ*_C_	*δ*_H_	*δ*_C_	*δ*_H_	*δ*_C_	*δ*_H_	*δ*_C_	*δ*_H_	*δ*_C_	*δ*_H_
3-*O-*										
Ara-1	103.8	4.52 d (5.0)	103.8	4.52 d (5.3)	103.8	4.52 d (5.3)	103.8	4.48 d (5.5)	103.8	4.48 d (5.5)
2	74.9	3.79	75.2	3.78	75.0	3.78	74.8	3.76	75.5	3.76
3	72.3	3.73	72.3	3.75	72.3	3.74	72.3	3.75	72.7	3.72
4	67.6	3.79	67.6	3.80	67.6	3.79	68.1	4.11	68.6	4.12
5	63.2	3.53 3.87	63.2	3.52 3.87	63.2	3.52 3.86	63.1	3.49 3.83	63.1	3.50 3.83
Rha I-1	100.1	5.21 s	100.1	5.22 s	100.1	5.21 s	100.0	5.18 s	100.3	5.18 s
2	70.4	4.09 s	70.4	4.09 s	70.4	4.09 s	70.4	4.05 s	70.8	4.06 s
3	80.7	3.87	80.7	3.86	80.7	3.85	80.6	3.84	81.2	3.83
4	71.4	3.59	71.4	3.60	71.4	3.60	71.3	3.56 t (9.4)	72.1	3.57
5	68.6	3.92	68.6	3.93	68.6	3.92	68.6	3.88	68.9	3.89
6	16.4	1.26 d (6.2)	16.5	1.26 d (6.1)	16.6	1.26 d (5.9)	16.6	1.22 d (5.9)	16.5	1.23 d (6.5)
Rha II-1	101.4	5.19 s	100.3	5.37 s	100.3	5.37 s	100.9	5.17 s	100.0	5.40 s
2	70.9	3.97 s	71.2	3.93 s	71.2	3.92 s	71.2	3.90 s	70.5	3.92 s
3	70.8	3.71	70.5	3.86	70.5	3.85	70.5	3.74	70.9	3.88
4	72.3	3.42	72.7	3.40	72.3	3.42	72.6	3.36	73.8	3.31
5	68.7	4.02 dq (9.6, 6.2)	68.1	4.39	68.0	4.37	68.1	4.22	68.0	4.46
6	16.4	1.27 d (6.2)	16.4	1.24 d (6.2)	16.5	1.24 d (6.2)	16.6	1.21 d (6.5)	16.5	1.22 d (5.9)
Xyl I-1	104.9	4.55 d (7.6)	104.9	4.53 d (7.4)	104.9	4.53 d (7.8)	104.9	4.49 d (7.6)	104.9	4.49 d (7.6)
2	73.9	3.36	74.0	3.37	73.7	3.35	73.8	3.34	73.8	3.34
3	74.4	3.52	74.4	3.53	74.4	3.52	74.0	3.48	75.1	3.50
4	76.9	3.72	76.8	3.73	76.9	3.71	76.9	3.69	77.8	3.69
5	63.2	3.34 4.06 dd (11.9, 5.4)	63.1	3.32 4.06 dd (11.7, 5.2)	63.1	3.32 4.06 dd (11.8, 5.3)	62.9	3.34 4.02 dd (11.9, 5.4)	63.1	3.31 4.02 dd (11.7, 5.3)
Xyl II-1			103.0	4.38 d (7.8)	103.0	4.38 d (7.7)	103.2	4.34 d (7.6)		
2			73.7	3.22	73.7	3.22	77.0	3.40		
3			76.6	3.28	76.7	3.30	75.0	3.76		
4			69.5	3.60	69.5	3.60	78.6	3.62		
5			65.4	3.15 3.91	65.4	3.14 3.90	63.1	3.32 4.11		
Xyl III-1							101.9	4.25 d (7.6)		
2							73.9	3.16		
3							76.6	3.27		
4							69.6	3.49		
5							65.6	3.14 3.88		
Glc I-1	101.7	4.42 d (7.7)	101.7	4.42 d (7.8)	101.7	4.41 d (7.7)	101.7	4.38 d (7.6)	102.5	4.38 d (7.6)
2	73.1	3.30	72.9	3.30	72.7	3.29	73.0	3.27	73.5	3.27
3	74.2	3.50	74.9	3.42	74.9	3.50	75.0	3.44	75.5	3.44
4	78.9	3.58	78.7	3.58	78.7	3.57	78.8	3.50	80.0	3.58
5	75.0	3.47	75.7	3.46	75.7	3.45	75.7	3.45	76.1	3.52
6	60.2	3.87 3.93	60.2	3.87 3.92	60.1	3.87 3.92	60.1	3.81 3.90	61.3	3.79 3.86
Glc II-1	103.0	4.43 d (7.8)	103.1	4.43 d (7.7)	103.1	4.42 d (7.7)			103,8	4.41 d (8.2)
2	73.9	3.35	76.7	3.44	75.8	3.47			73.5	3.43
3	82.7	3.53	76.4	3.54	76.4	3.54			74.5	3.45
4	68.6	3.38	76.9	3.74	76.9	3.73			77.0	3.78
5	76.5	3.32	75.8	3.48	75.8	3.44			75.5	3.52
6	61.0	3.92, nd	59.8	3.93, nd	59.6	3.93, nd			60.3	3.91, nd
28-*O*-										
Glc III-1	94.4	5.38 d (8.1)	94.4	5.38 d (8.1)			94.3	5.34 d (8.2)	94.8	5.34 d (8.2)
2	72.4	3.36	72.5	3.36			72.5	3.36	73.0	3.35
3	76.7	3.44	74.7	3.55			74.9	3.50	74.9	3.54
4	69.5	3.46	69.5	3.45			69.6.	3.42	70.5	3.44
5	76.2	3.53	76.7	3.43			76.6	3.41	76.8	3.42
6	68.1	3.79 4.15 dd (11.9, 1.5)	68.1	3.80 4.14 dd (11.8, 1.9)			68.1	3.89 4.11	68.6	3.83 4.12 dd (10.9, 1.5)
Glc IV-1	103.2	4.37 d (7.7)	103.2	4.37 d (7.4)			103.2	4.33 d (7.6)	103.2	4.33 d (7.6)
2	73.7	3.23 dd (9.0, 8.0)	73.6	3.23			73.0	3.20 dd (8.8, 7.6)	74.4	3.20 dd (9.4, 7.6)
3	76.7	3.39	76.7	3.38			76.8	3.35	77.1	3.37
4	70.1	3.32	70.1	3.32			71.0	3.28	71.0	3.29
5	76.7	3.27	76.6	3.30			76.8	3.25	77.1	3.25
6	61.3	3.70 3.87	61.3	3.69 dd (12.0, 5.6) 3.88			61.3	3.67 3.87	62.0	3.68 dd (11.7, 5.3) 3.86

* Overlapped signals are reported without designated multiplicity. *δ* in ppm; *J* in parentheses in Hz. Nd: not determined.

## Supplementary Materials

The following supporting information can be downloaded at: https://www.mdpi.com/article/10.3390/molecules28114329/s1. The following are available online: Figures S1–S5. HSQC, HMBC, COSY, ROESY, and TOCSY spectra of compound **1**; Figures S6–S10. HSQC, HMBC, COSY, ROESY, and TOCSY spectra of compound **2**; Figures S11–S15. HSQC, HMBC, COSY, ROESY, and TOCSY spectra of compound **3**; Figures S16–S20. HSQC, HMBC, COSY, ROESY, and TOCSY spectra of compound **4**; Figures S21–S25. HSQC, HMBC, COSY, ROESY, and TOCSY spectra of compound **5**.

## Sample Availability

Samples of crude extracts are available from the authors.
