# Supplementary material for: Oleanolic Acid Glycosides from Scabiosa caucasica and Scabiosa ochroleuca: Structural Analysis and Cytotoxicity
Source: Molecules. 2023 May 25;28(11):4329. doi: 10.3390/molecules28114329 (PMC10254451; doi:10.3390/molecules28114329)
Supplement: Supplementary file 1 [file molecules-28-04329-s001.zip › molecules-2361883-supplementary.pdf]

Supplementary Materials

Oleanolic Acid Glycosides from *Scabiosa caucasica* and *Scabiosa ochroleuca* : Structural Analysis and Cytotoxicity

Samvel Nazaryan [1,2], Antoine Bruguière [1], Nelli Hovhannisyan [2], Tomofumi Miyamoto [3], Alexandre M. M. Dias [4], Pierre-Simon Bellaye [5], Bertrand Collin [6], Loïc Briand [1] and Anne-Claire Mitaine-Offer [1,*]

1 Center for Taste and Feeding Behavior (CSGA), CNRS, INRAE, Agro Institute, CNRS, INRAE, Institut Agro, Université de Bourgogne, Dijon, France; samvel.nazaryan@univ-fcomte.fr (S.N.); antoine.bruguiere@u-bourgogne.fr (A.B.); loic.briand@inrae.fr (L.B.)

2 Yerevan State University (YSU), Yerevan, Armenia; nellihovhannisyannh@gmail.com

3 Graduate School of Pharmaceutical Sciences, Kyushu University, Fukuoka, Japan; miyamoto@phar.kyushu-u.ac.jp

4 Plateforme d'Imagerie et de Radiothérapie Précliniques, Service de Médecine Nucléaire, Centre Georges-François Leclerc, Dijon, France; aldias@cgfl.fr

5 Plateforme d'Imagerie et de Radiothérapie Précliniques, Service de Médecine Nucléaire, Centre Georges-François Leclerc, UMR INSERM, Université de Bourgogne, Institut Agro 1231, Dijon, France; psbellaye@cgfl.fr

6 Plateforme d'Imagerie et de Radiothérapie Précliniques, Service de Médecine Nucléaire, Centre Georges-François Leclerc, ICMUB, UMR CNRS, Université de Bourgogne 6302, Dijon, France; bertrand.collin@u-bourgogne.fr

* Correspondence: anne-claire.offer@u-bourgogne.fr

Table of figures

Figure S1 – HSQC spectra of compound 1 3
Figure S2 – HMBC spectra of compound 1 4
Figure S3 – COSY spectra of compound 1 5
Figure S4– ROESY spectra of compound 1 6
Figure S5 -TOCSY spectra of compound 1 7
Figure S6 – HSQC spectra of compound 2 8
Figure S7 – HMBC spectra of compound 2 9
Figure S8 – COSY spectra of compound 2 10
Figure S9 – ROESY spectra of compound 2 11
Figure S10 – TOCSY spectra of compound 2 12
Figure S11 – HSQC spectra of compound 3 13
Figure S12 – HMBC spectra of compound 3 14
Figure S13 – COSY spectra of compound 3 15
Figure S14 – ROESY spectra of compound 3 16
Figure S15 – TOCSY spectra of compound 3 17
Figure S16 – HSQC spectra of compound 4 18
Figure S17 – HMBC spectra of compound 4 19
Figure S18 – COSY spectra of compound 4 20
Figure S19 – ROESY spectra of compound 4 21
Figure S20 – TOCSY spectra of compound 4 22
Figure S21 – HSQC spectra of compound 5 23
Figure S22 – HMBC spectra of compound 5 24
Figure S23 – COSY spectra of compound 5 25
Figure S24 – ROESY spectra of compound 5 26
Figure S25 – TOCSY spectra of compound 5 27

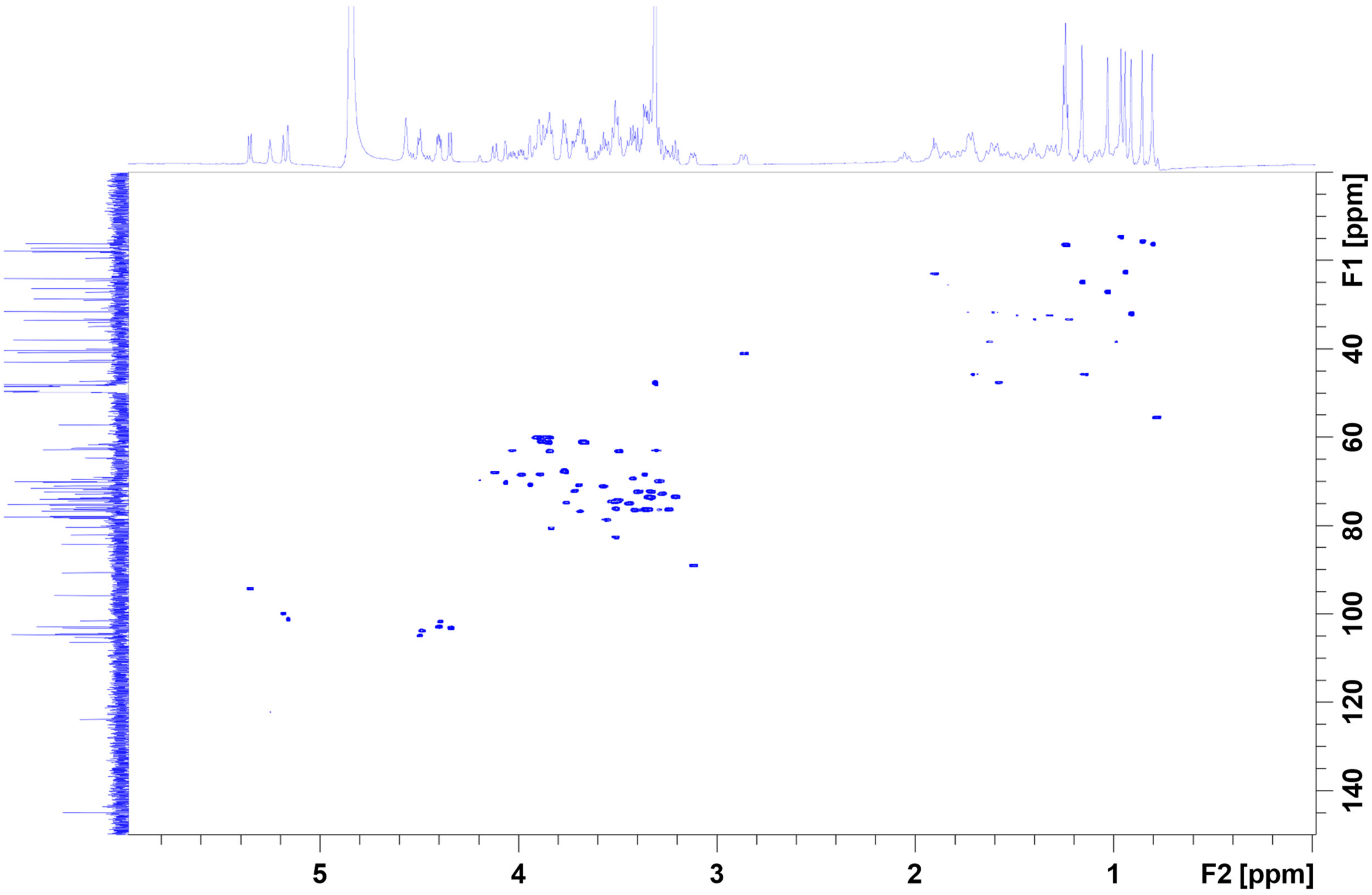

Figure S1 – HSQC spectra of compound 1

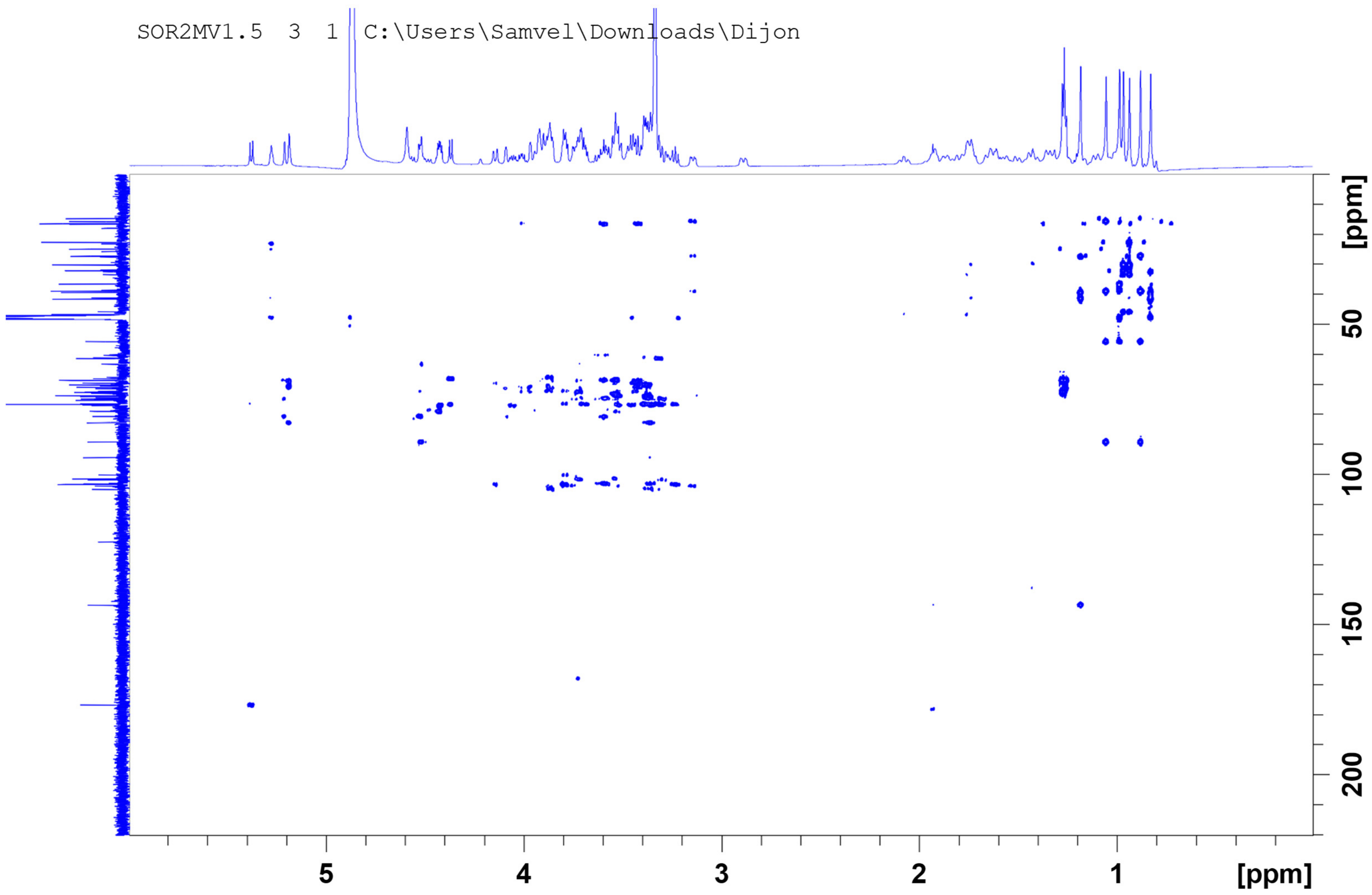

Figure S2 – HMBC spectra of compound 1

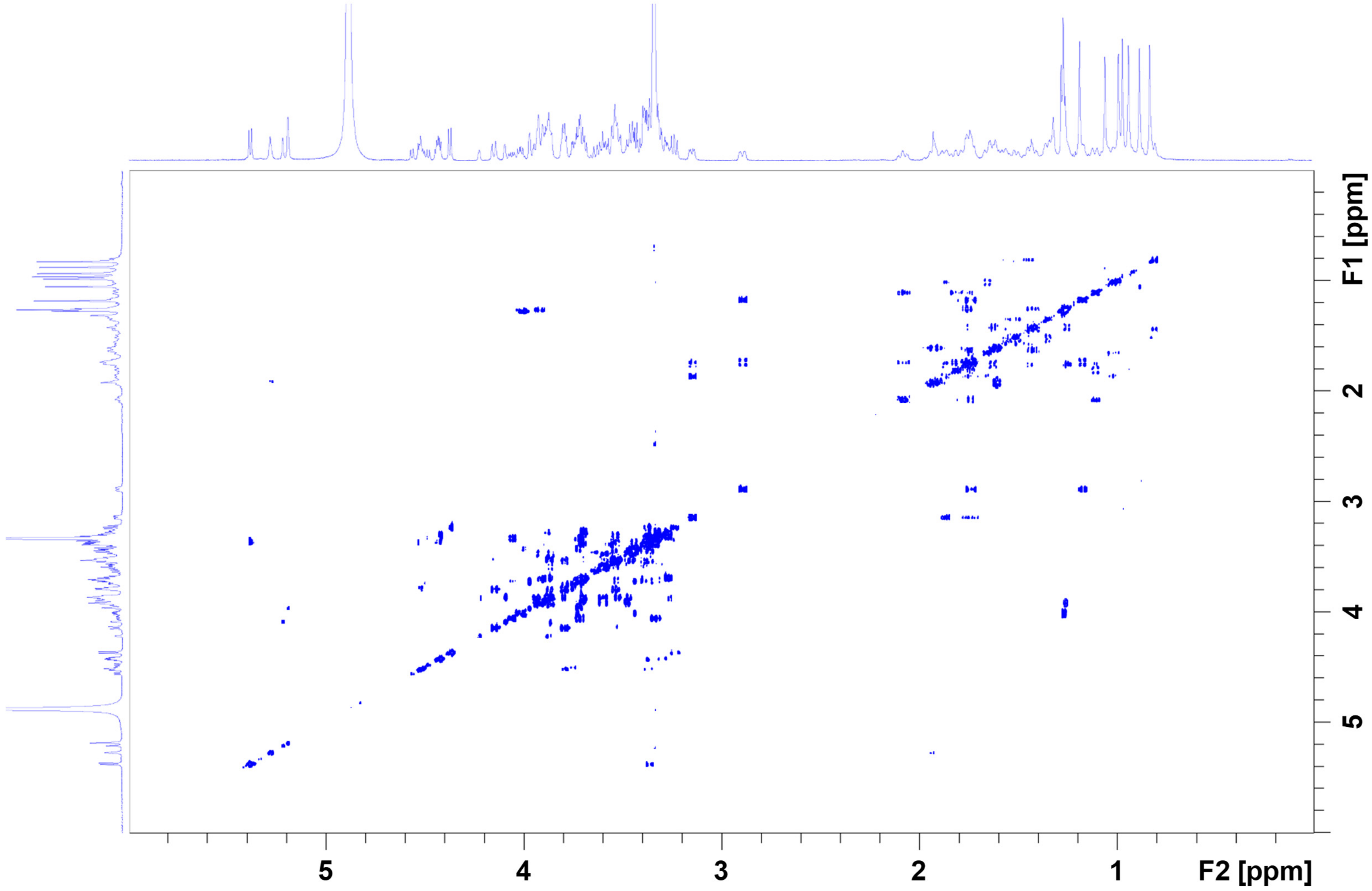

Figure S3 – COSY spectra of compound 1

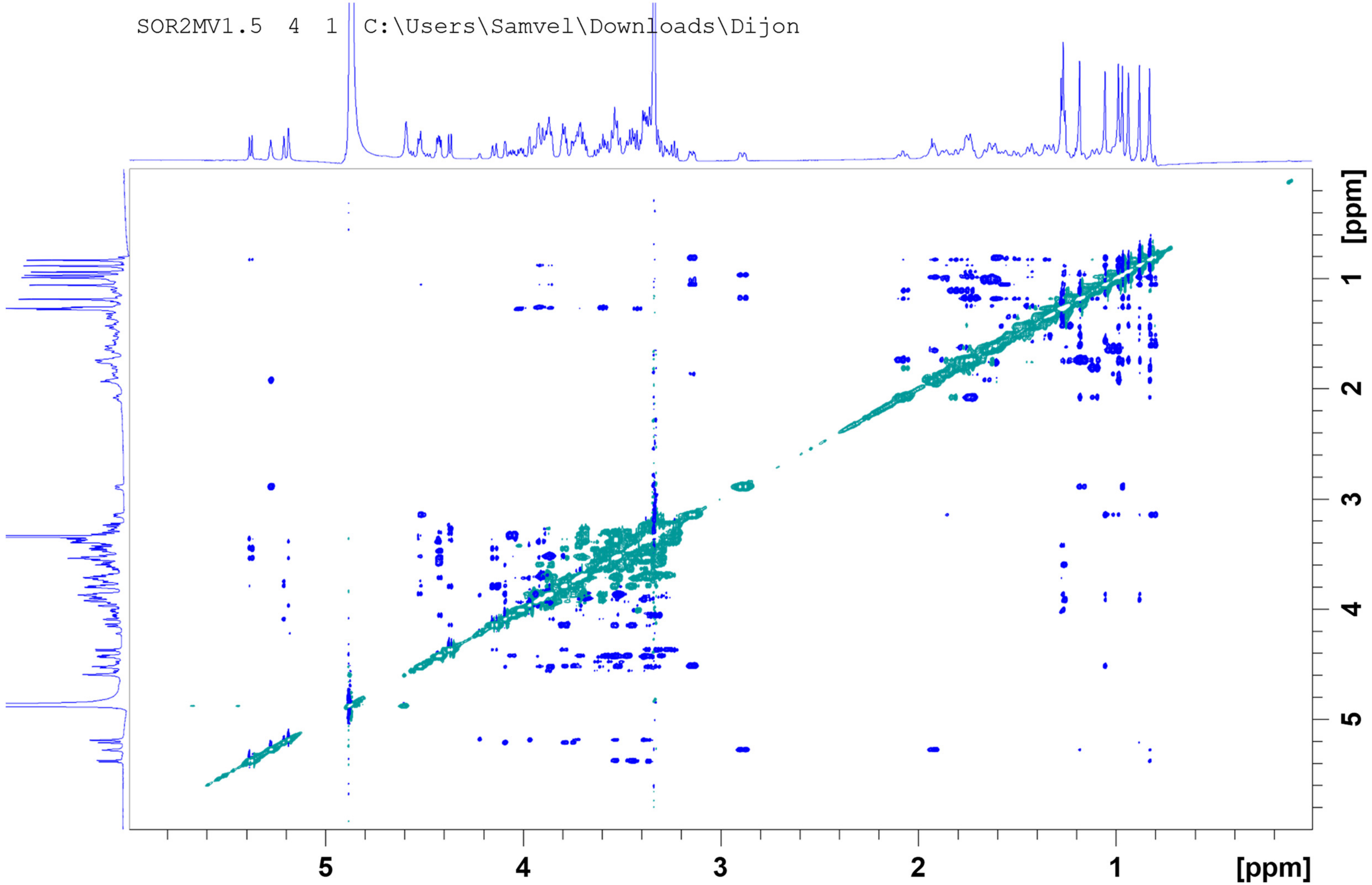

Figure S4 – ROESY spectra of compound 1

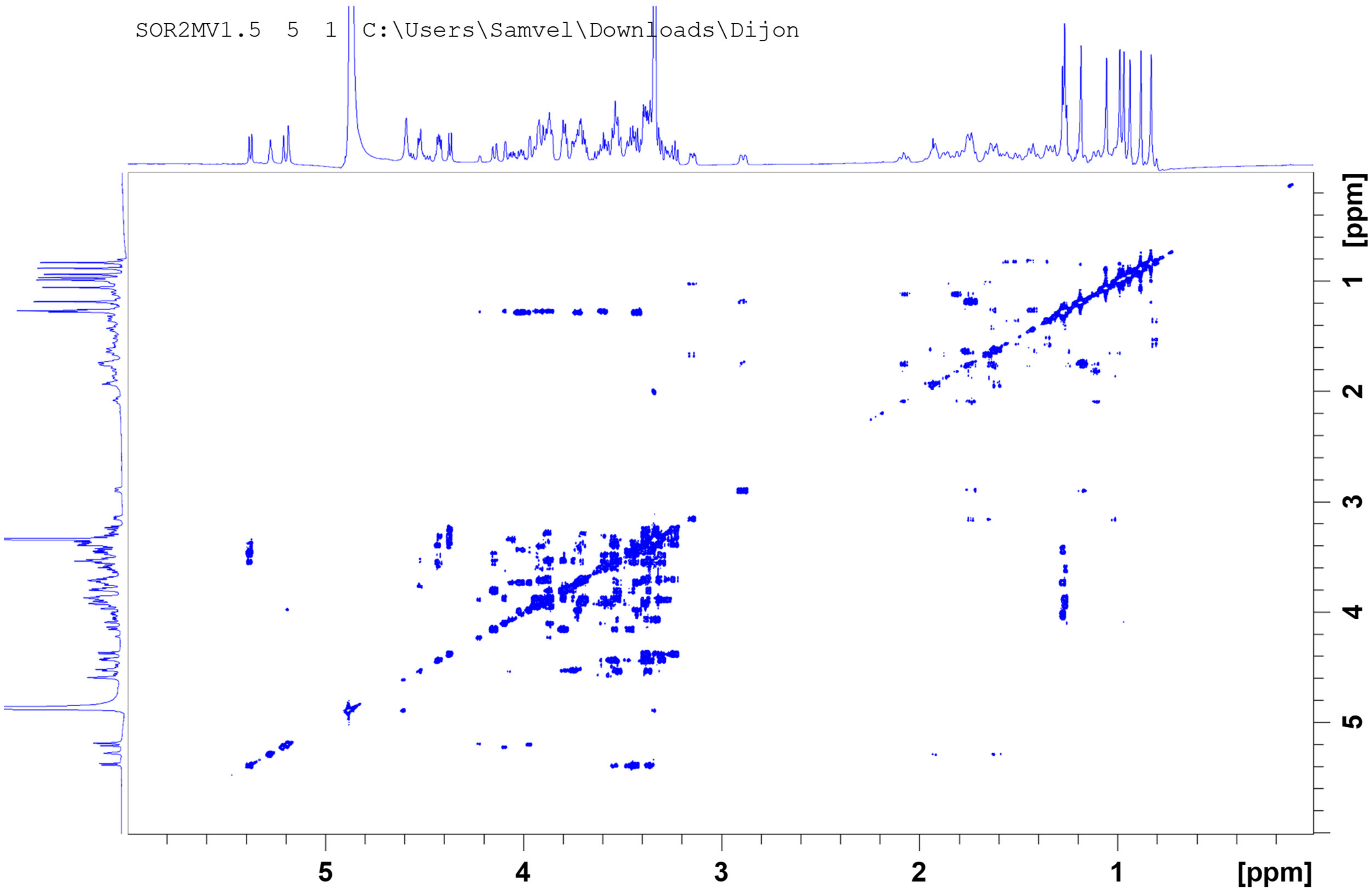

Figure S5 – TOCSY spectra of compound 1

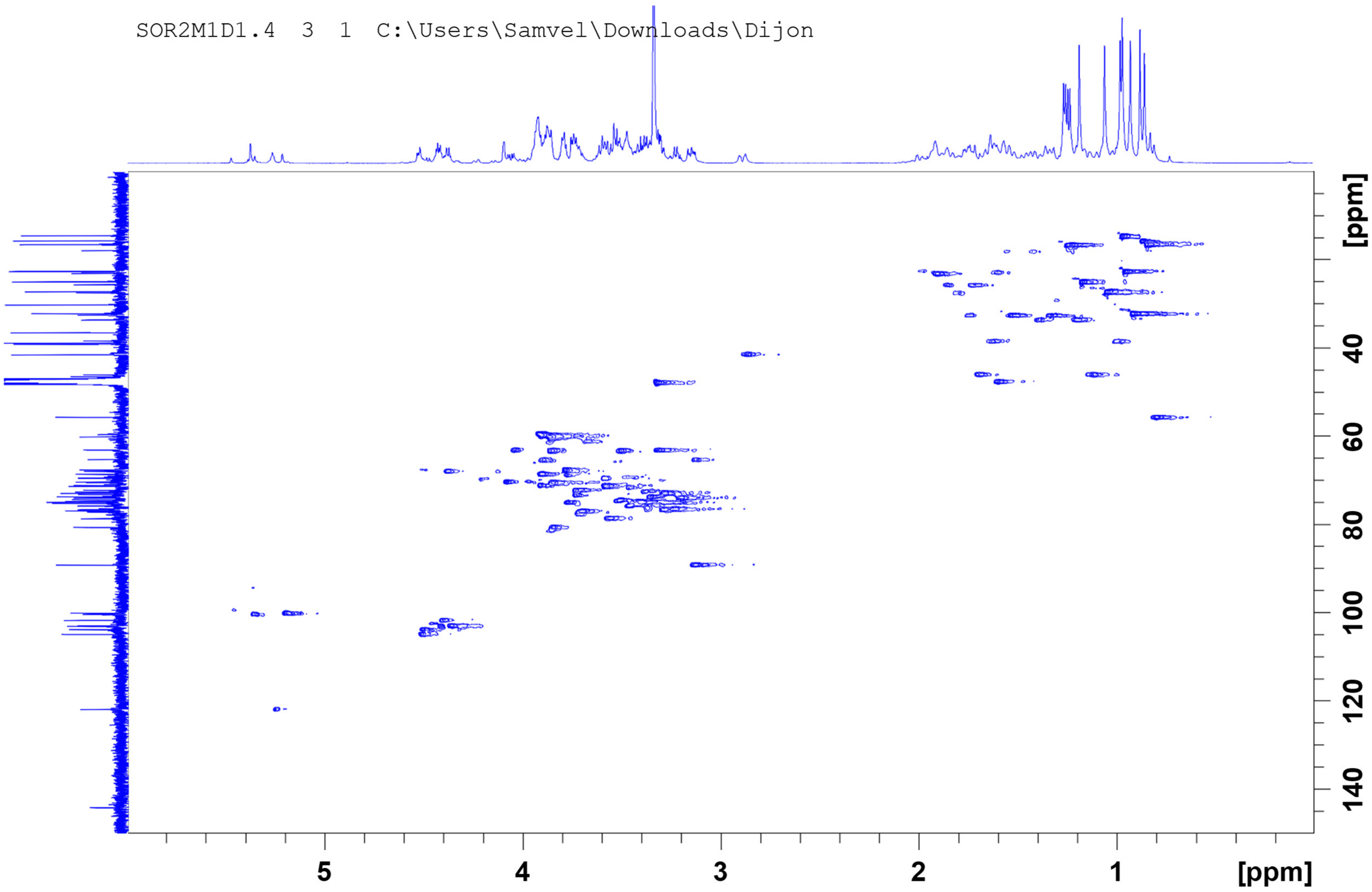

Figure S6 – HSQC spectra of compound 2

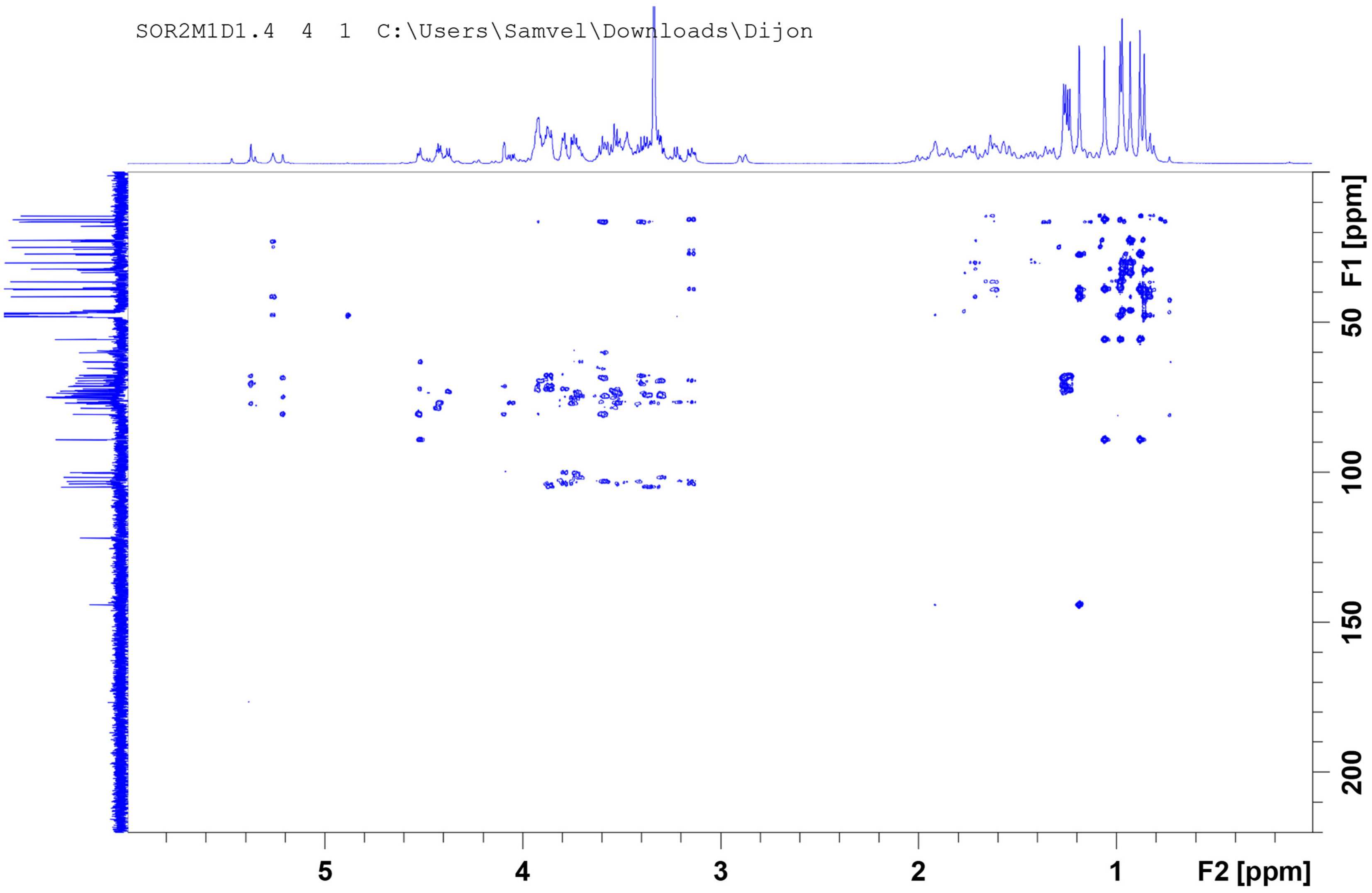

Figure S7 – HMBC spectra of compound 2

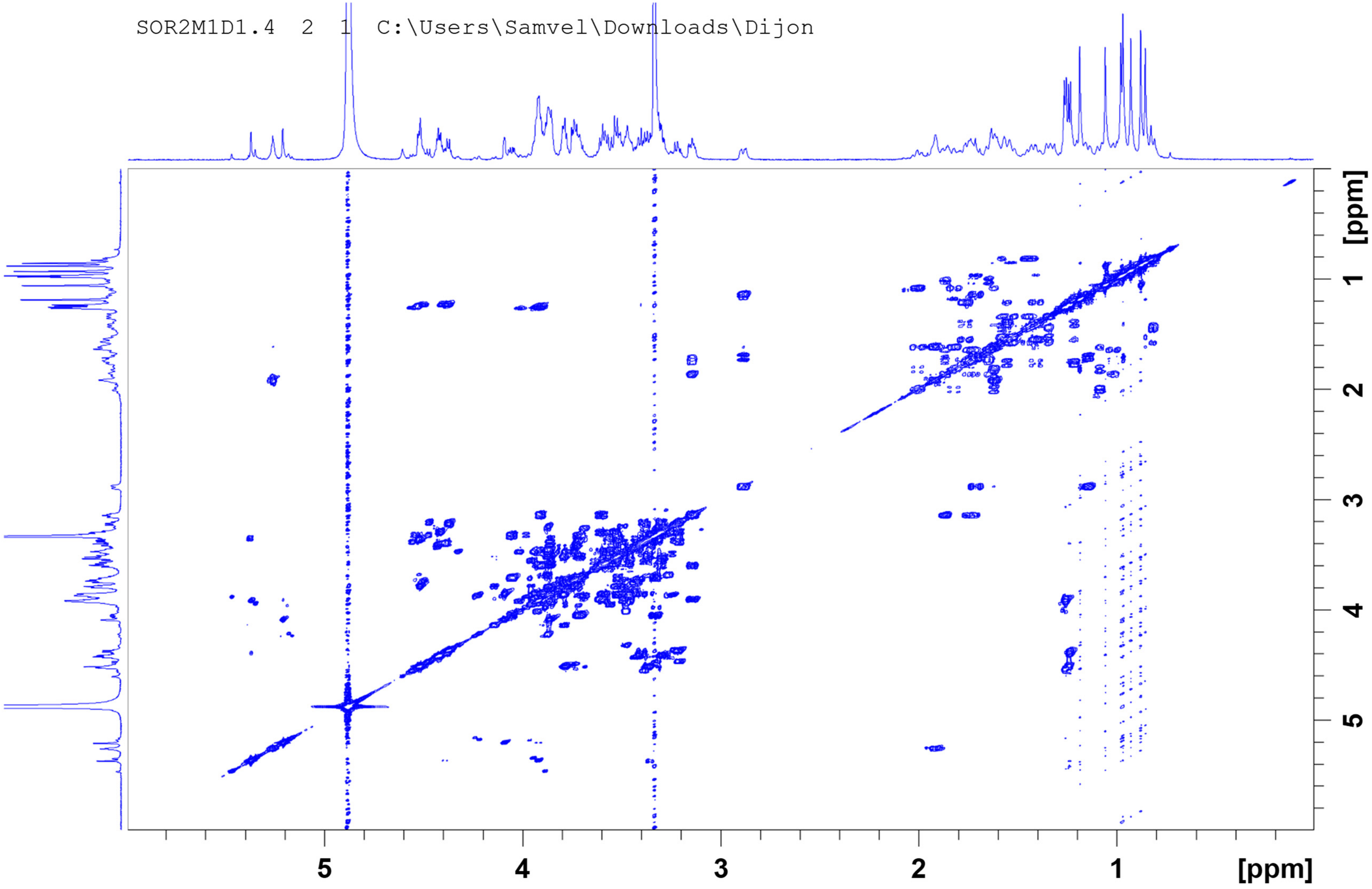

Figure S8 – COSY spectra of compound 2

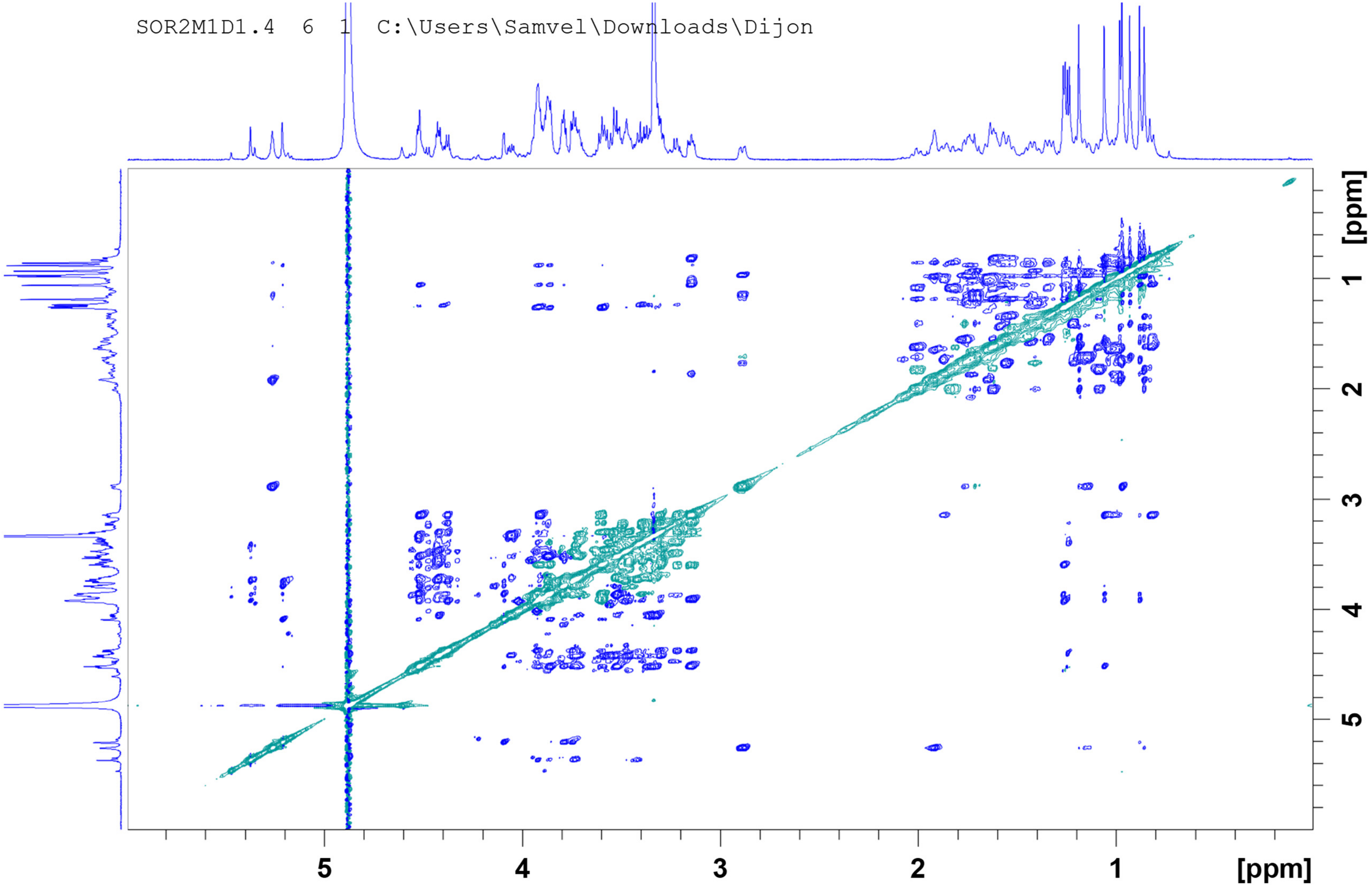

Figure S9 – ROESY spectra of compound 2

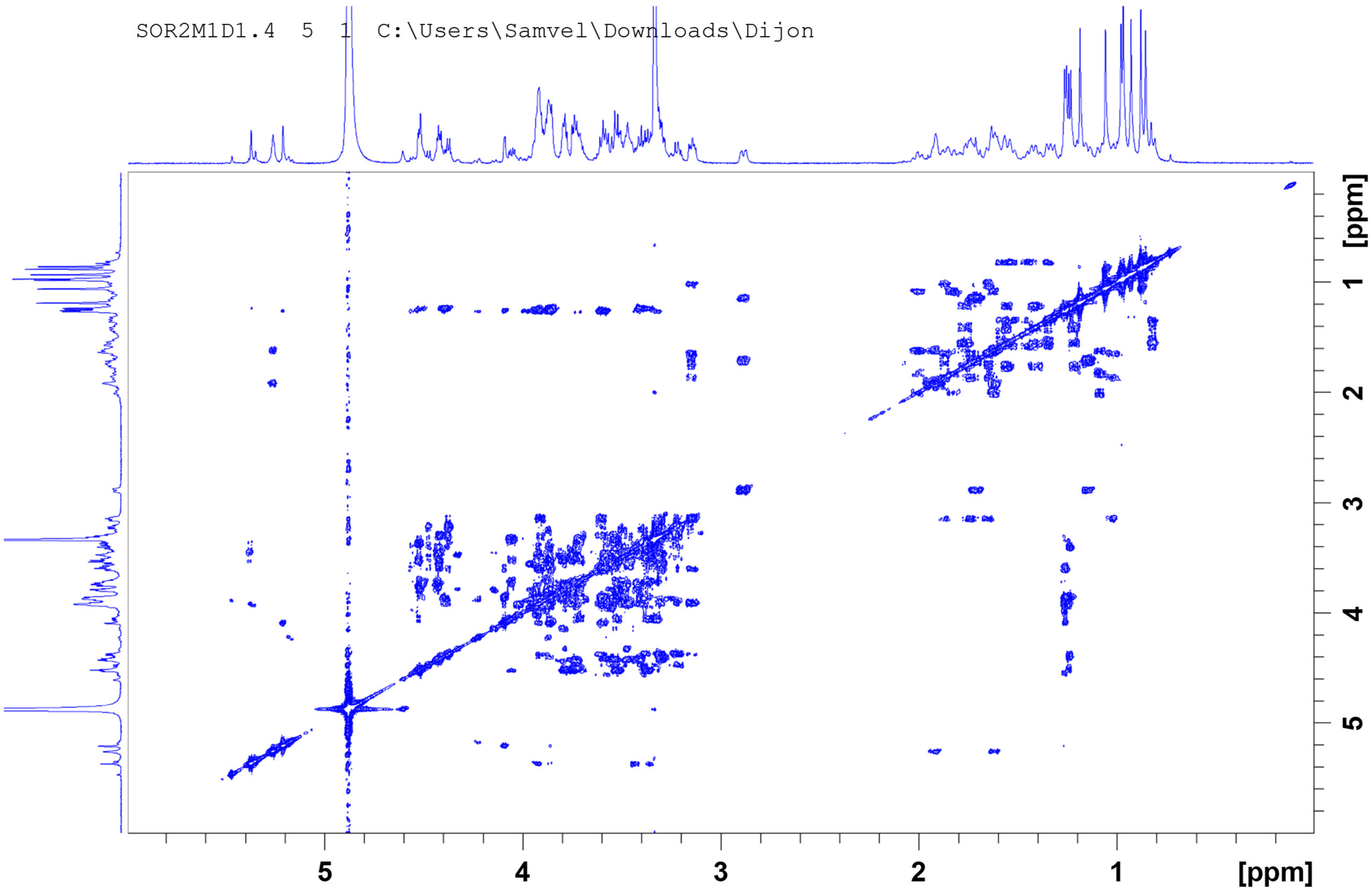

Figure S10 – TOCSY spectra of compound 2

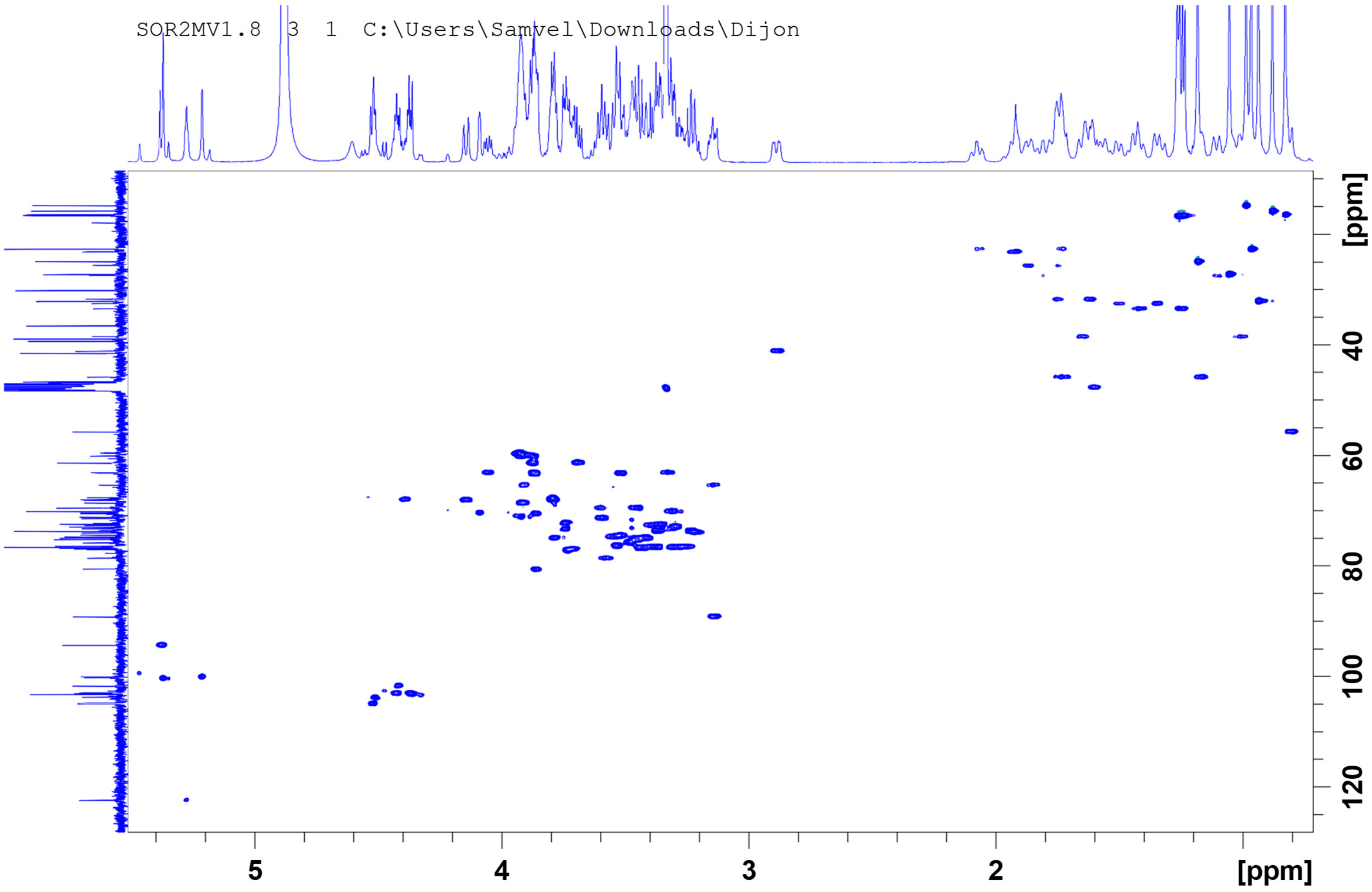

Figure S11 – HSQC spectra of compound 3

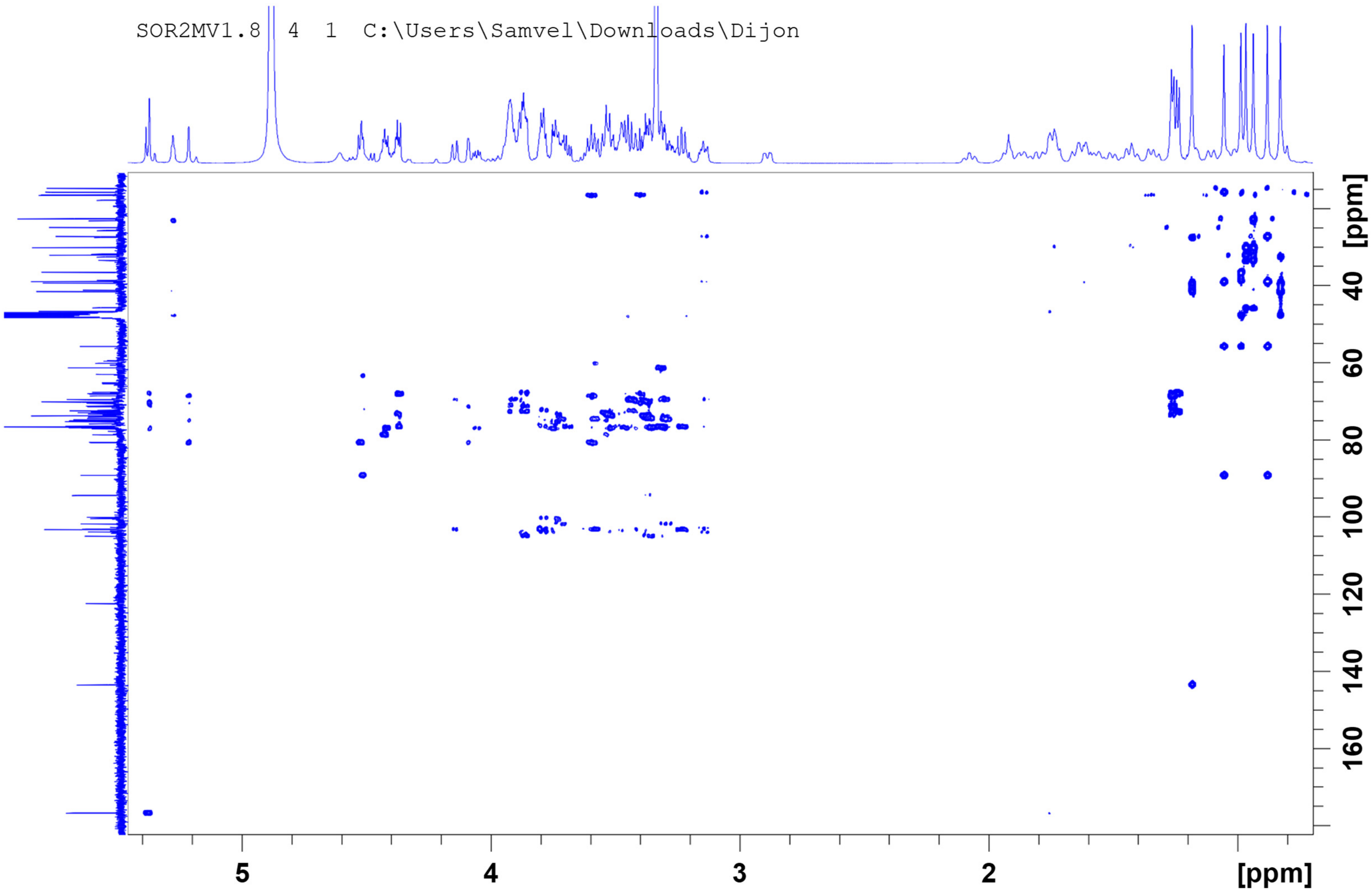

Figure S12 – HMBC spectra of compound 3

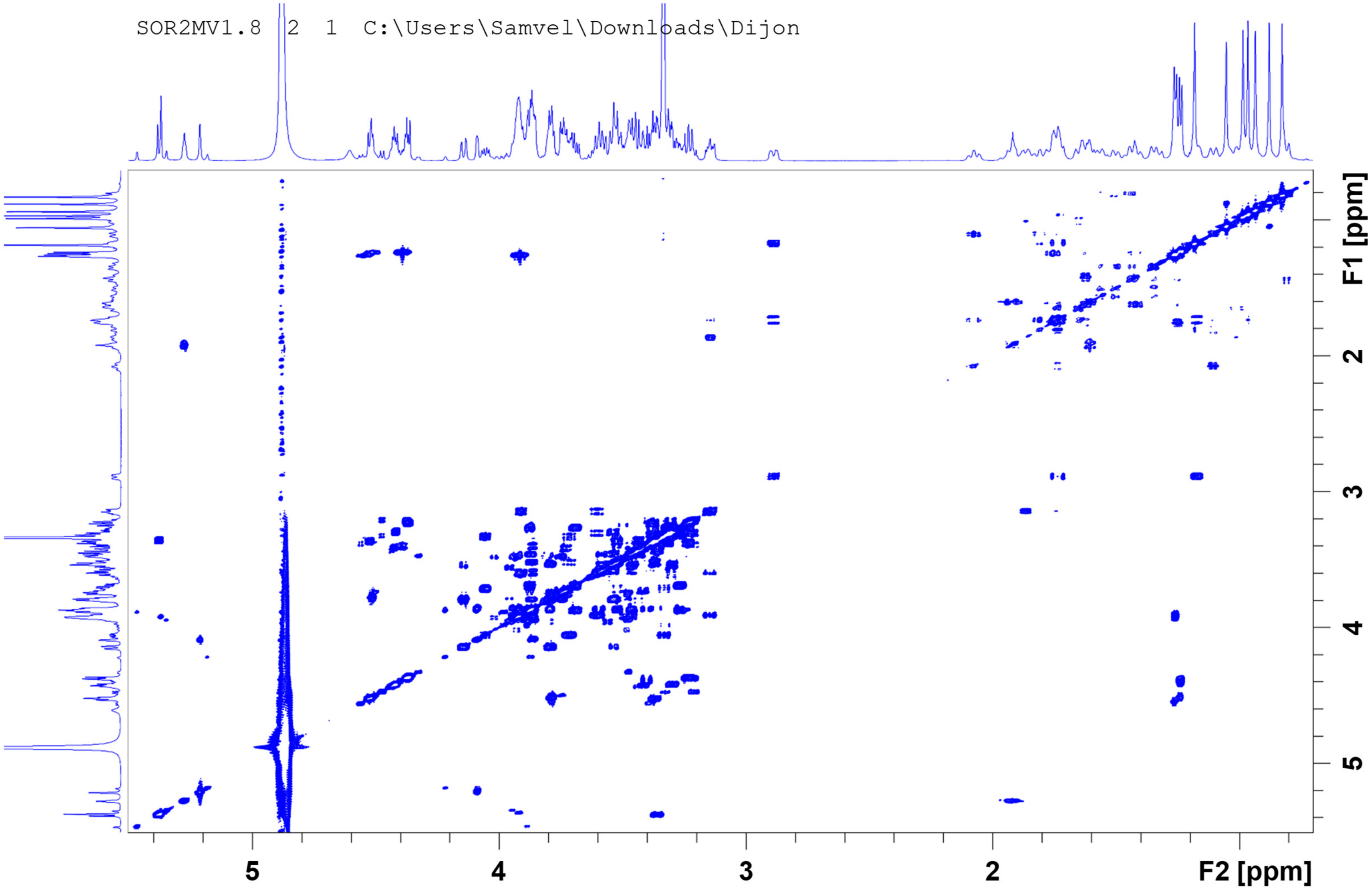

Figure S13 – COSY spectra of compound 3

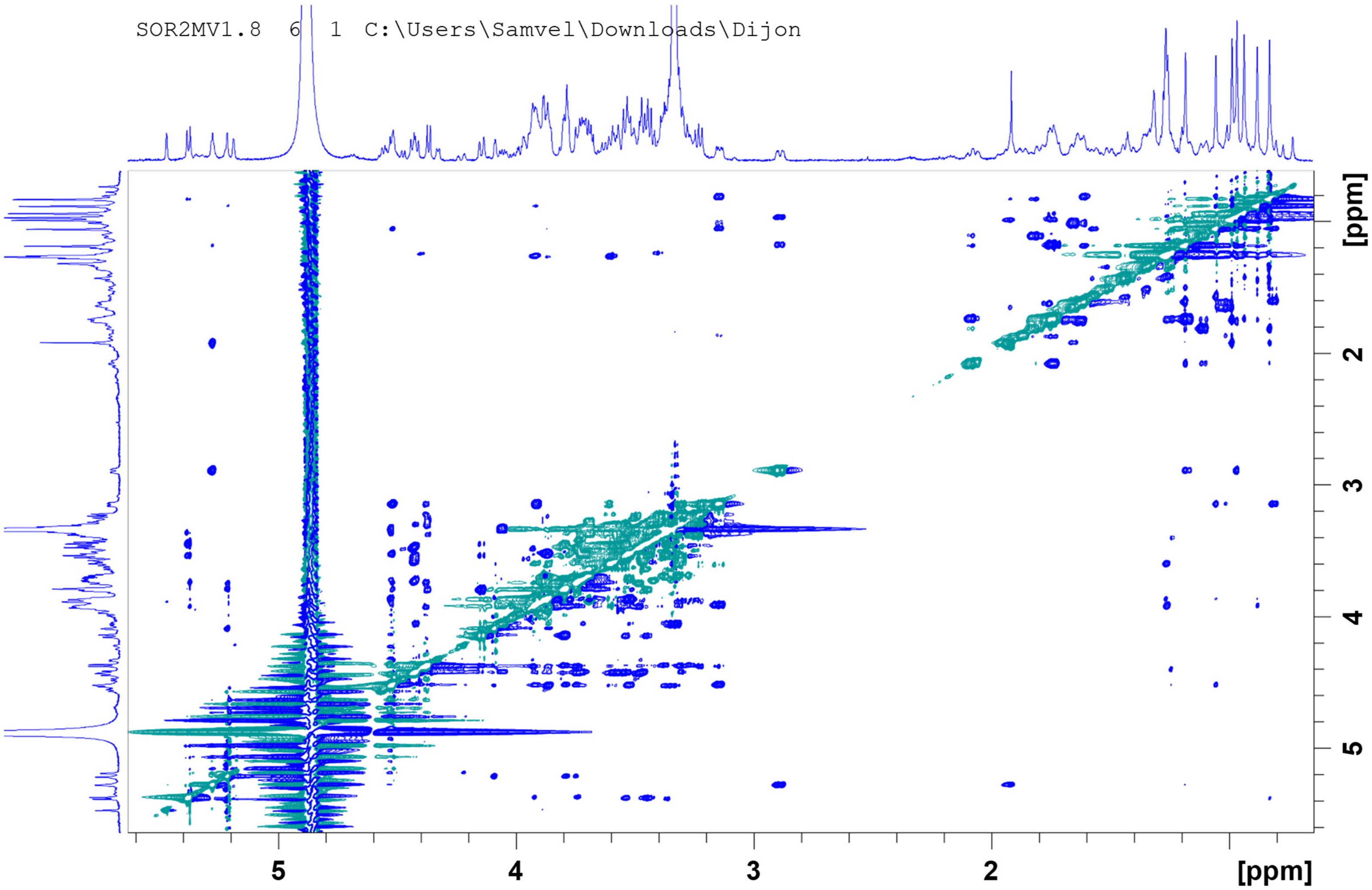

Figure S14 – ROESY spectra of compound 3

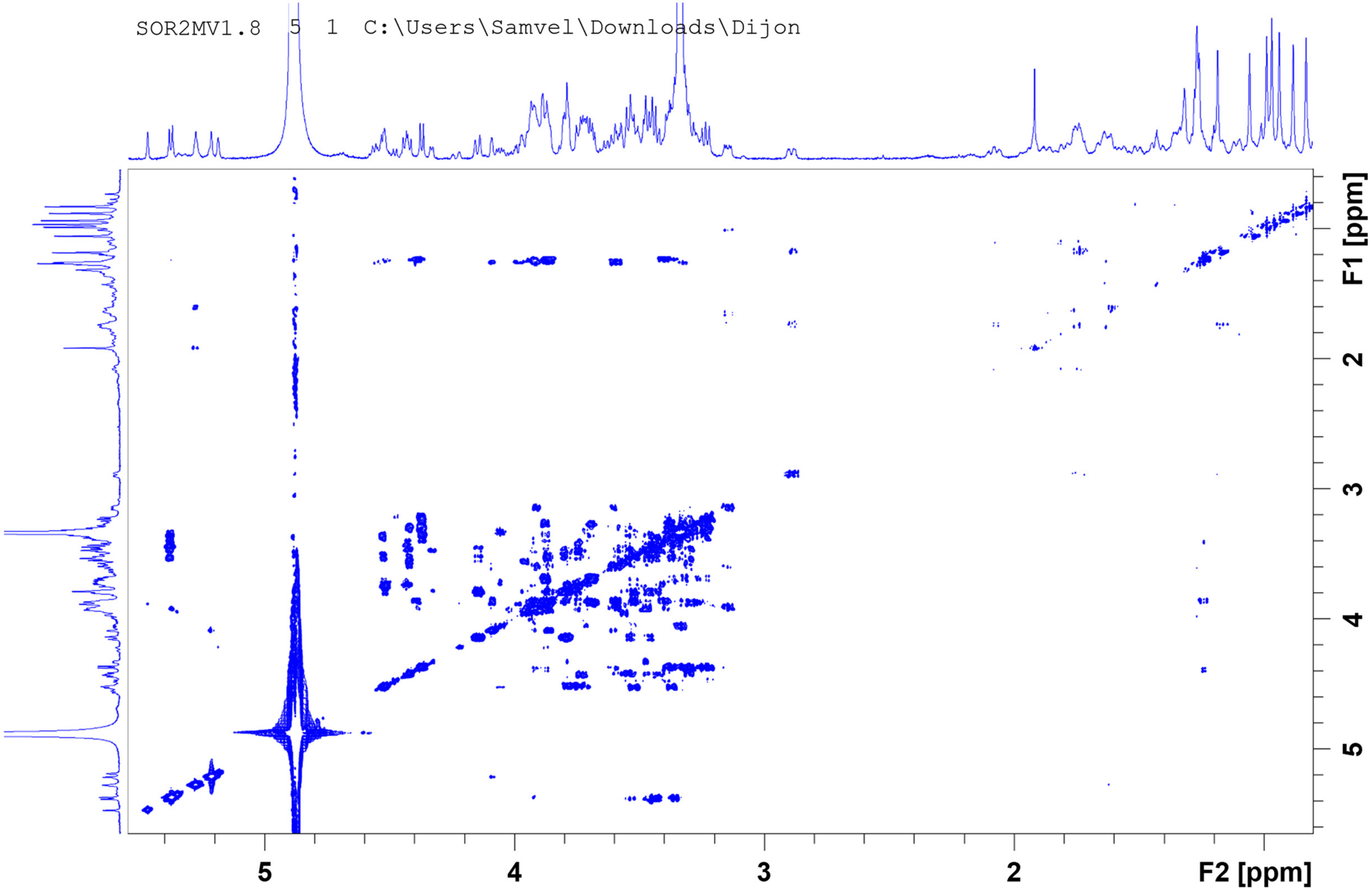

Figure S15 – TOCSY spectra of compound 3

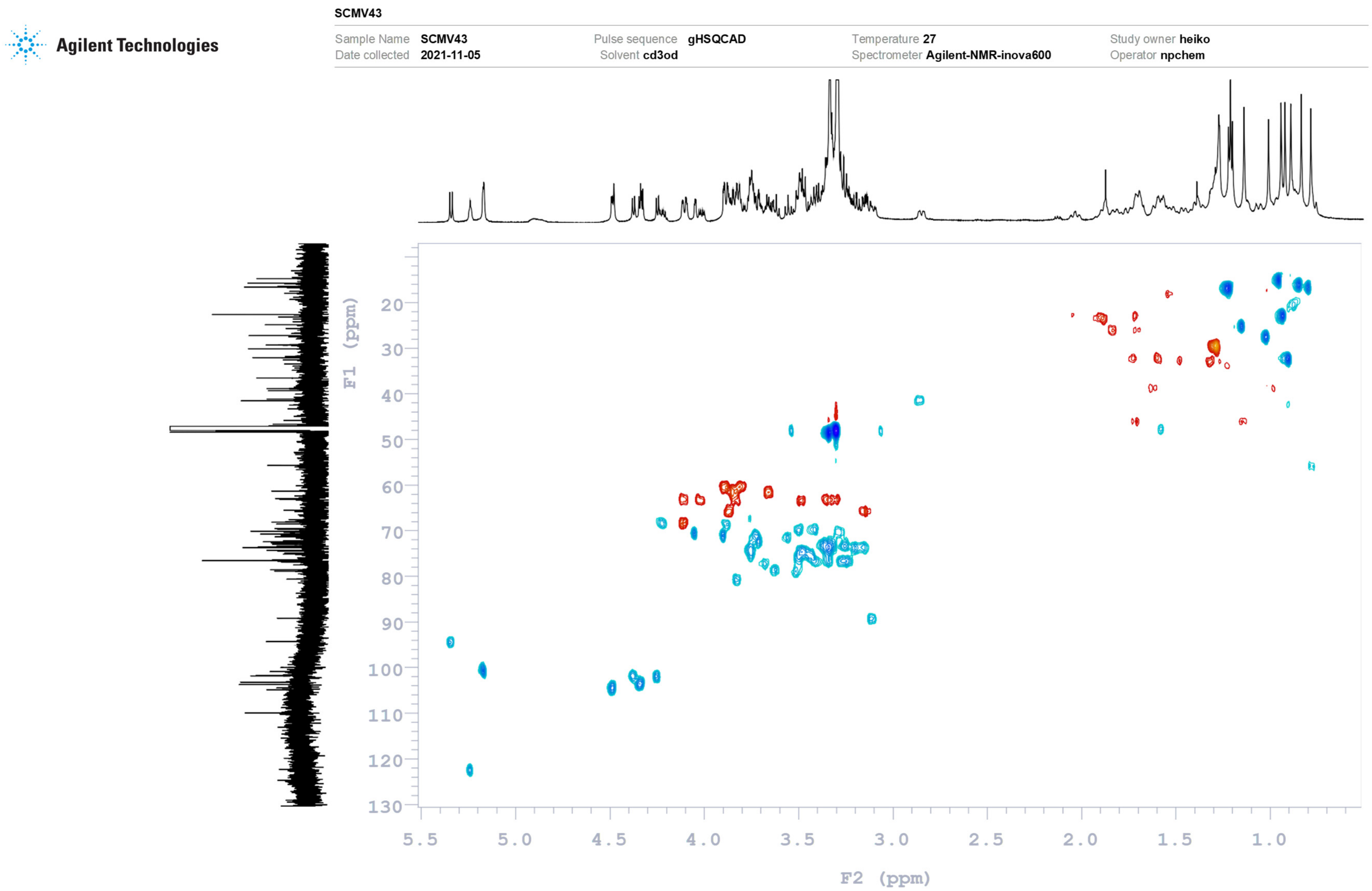

Figure S16 – HSQC spectra of compound 4

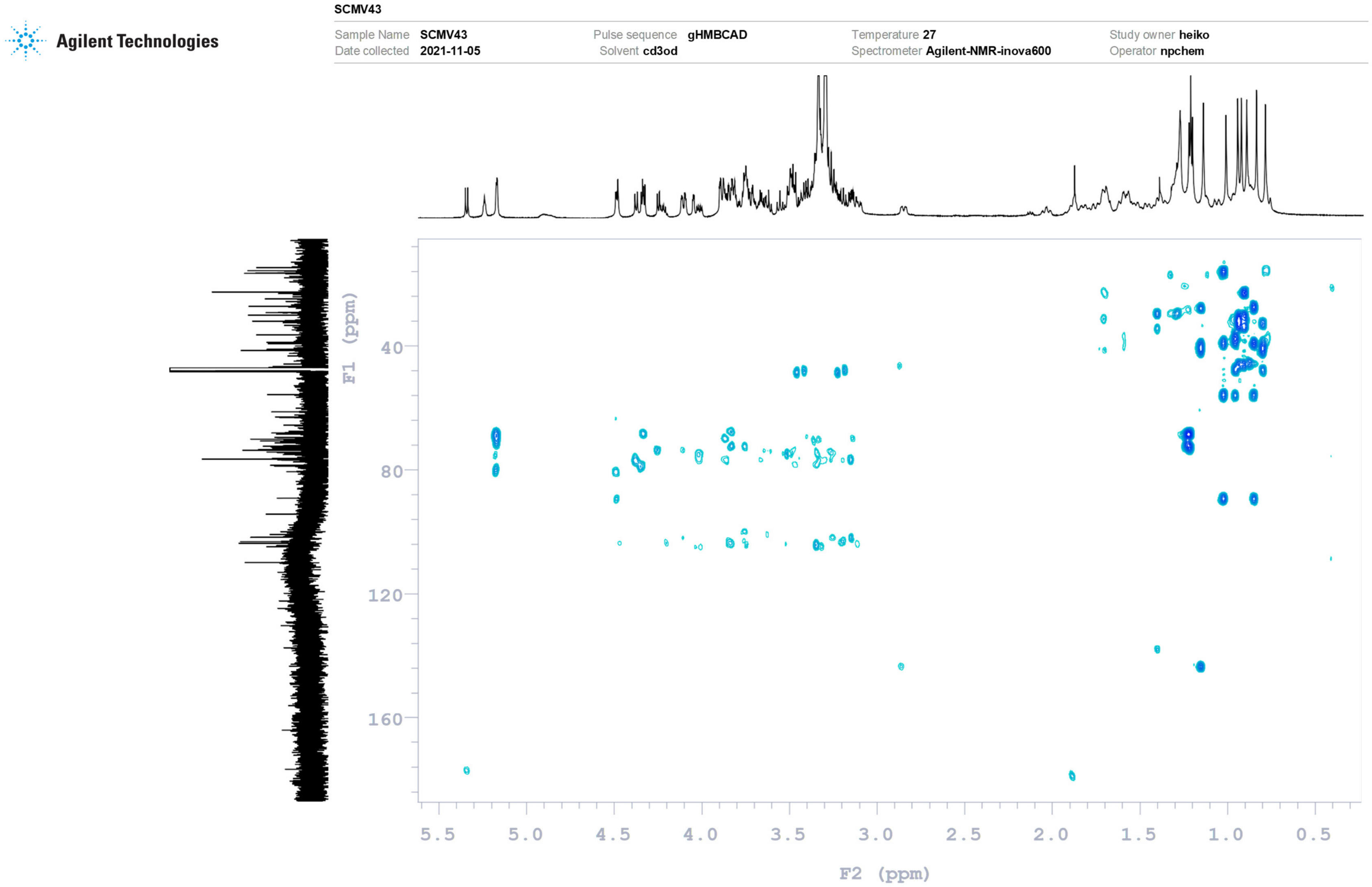

Figure S17 – HMBC spectra of compound 4

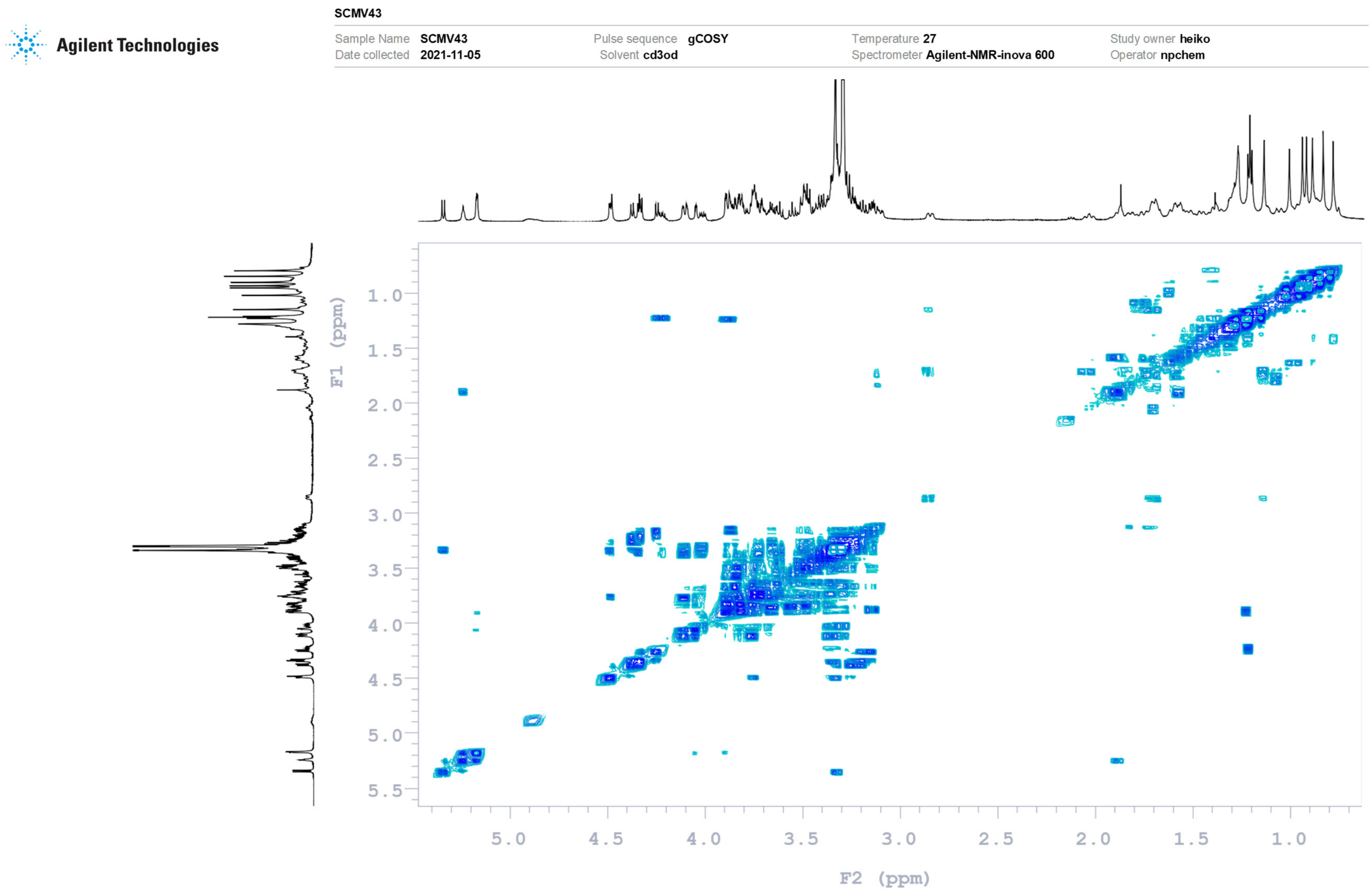

Figure S18 – COSY spectra of compound 4

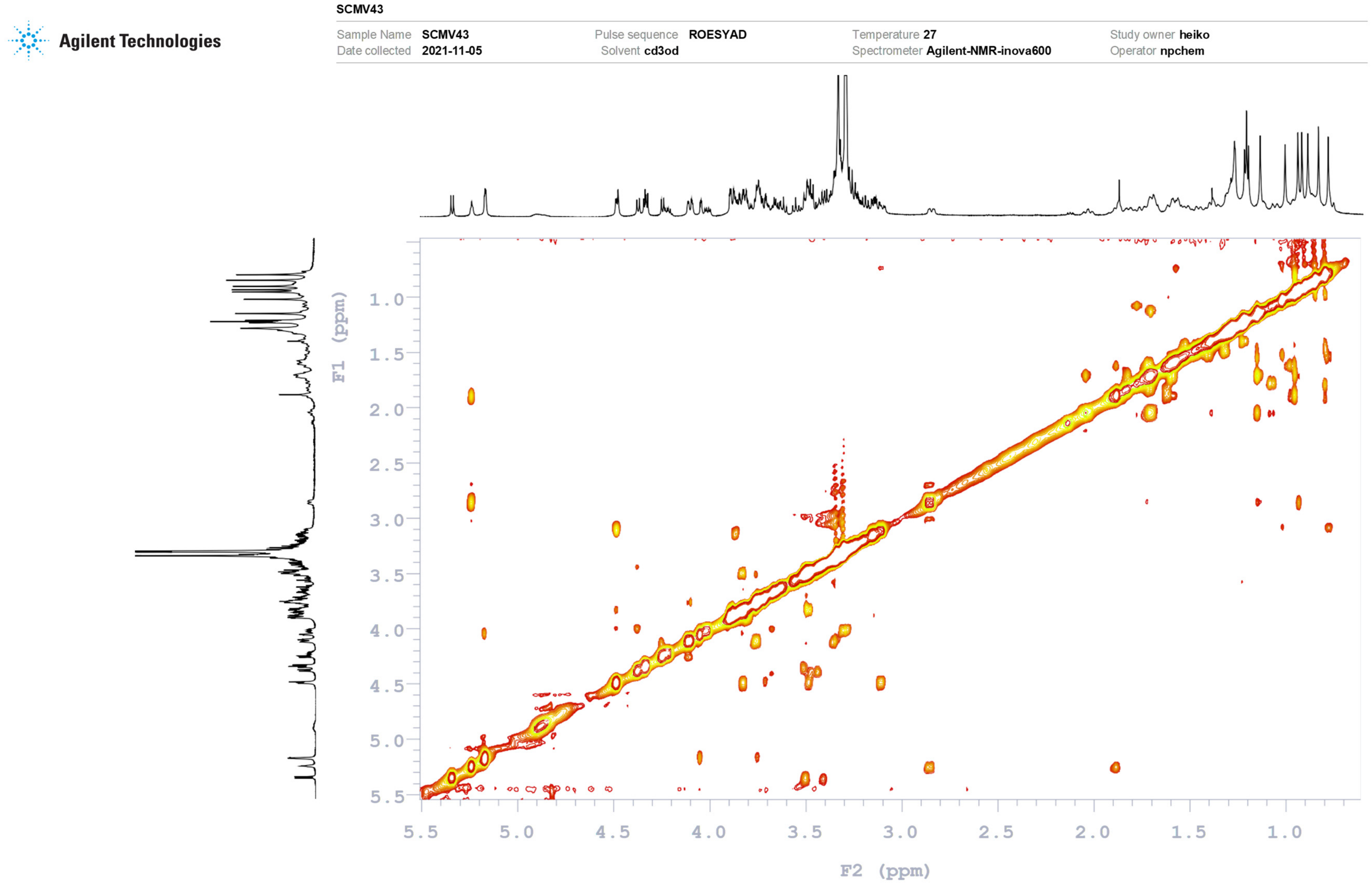

Figure S19 – ROESY spectra of compound 4

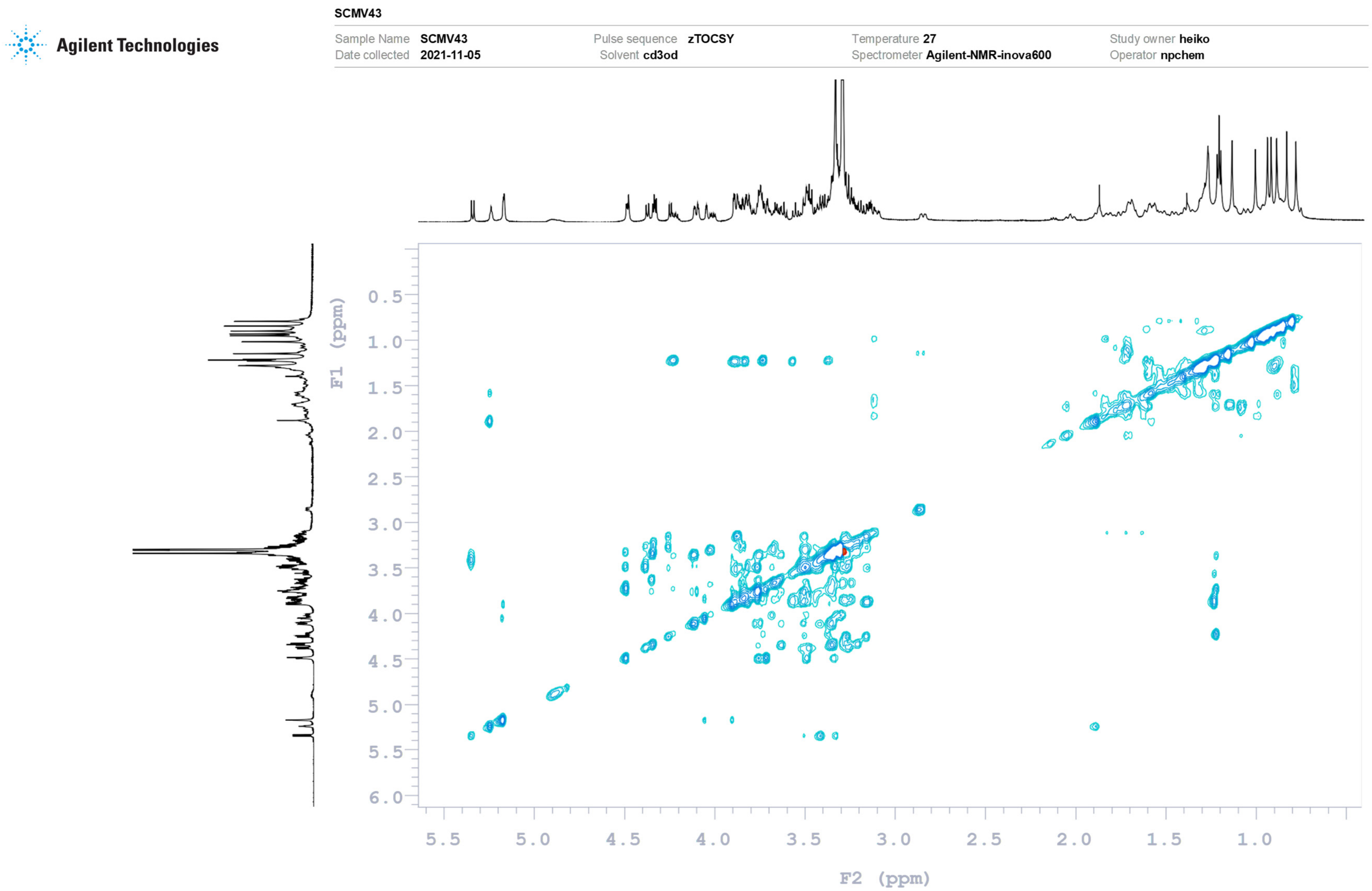

Figure S20 – TOCSY spectra of compound 4

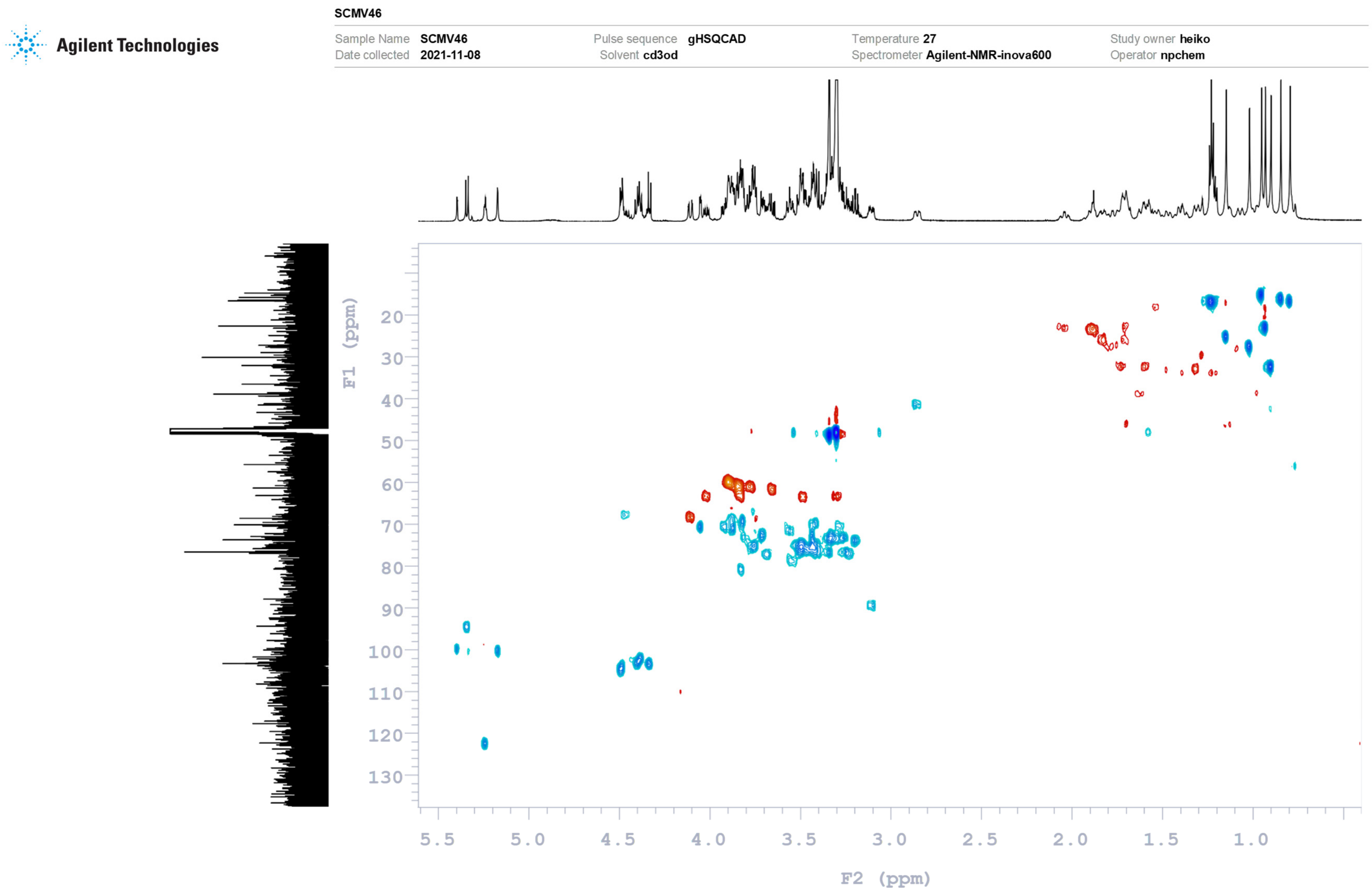

Figure S21 – HSQC spectra of compound 5

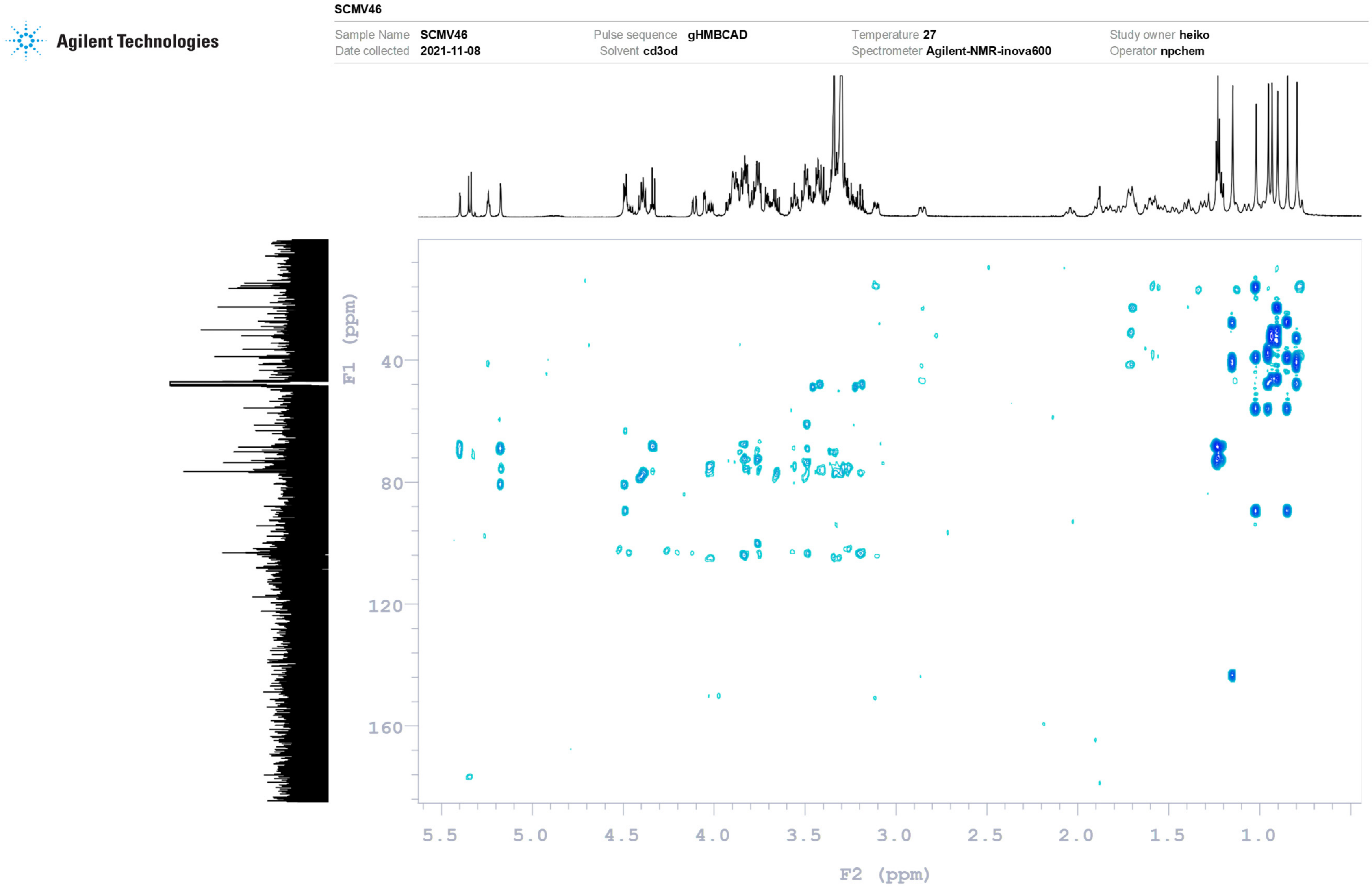

Figure S22 – HMBC spectra of compound 5

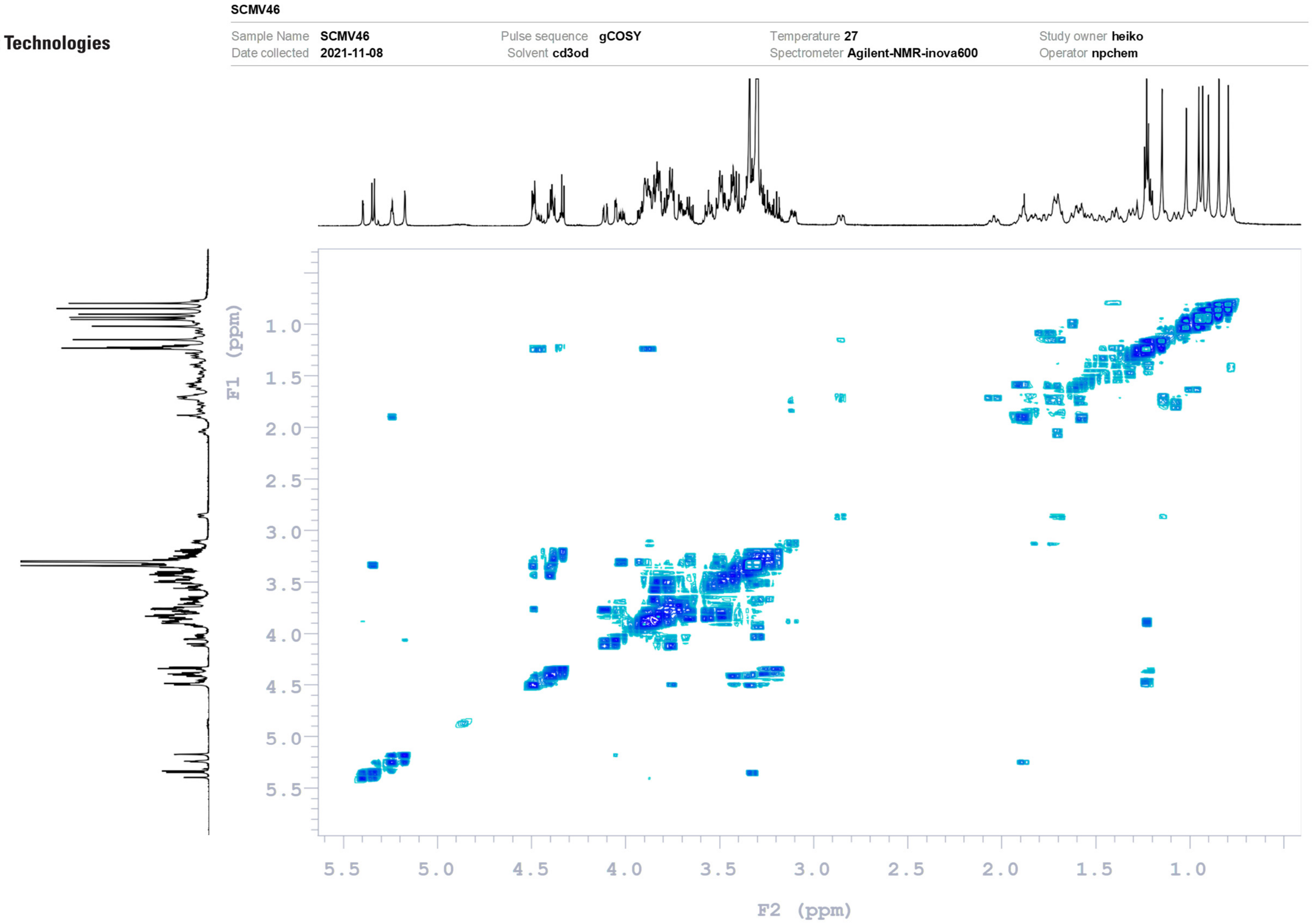

Figure S23 – COSY spectra of compound 5

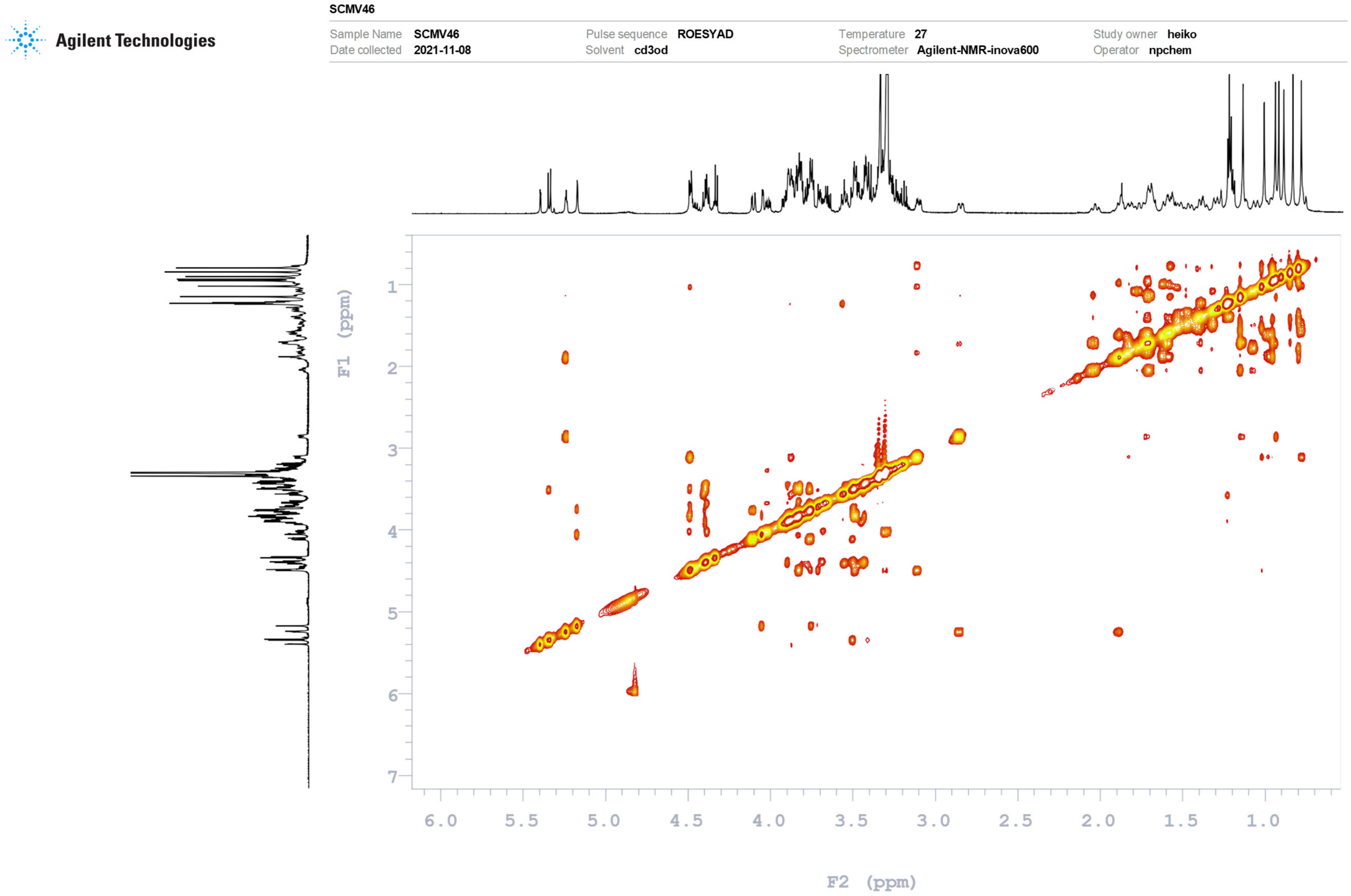

Figure S24 – ROESY spectra of compound 5

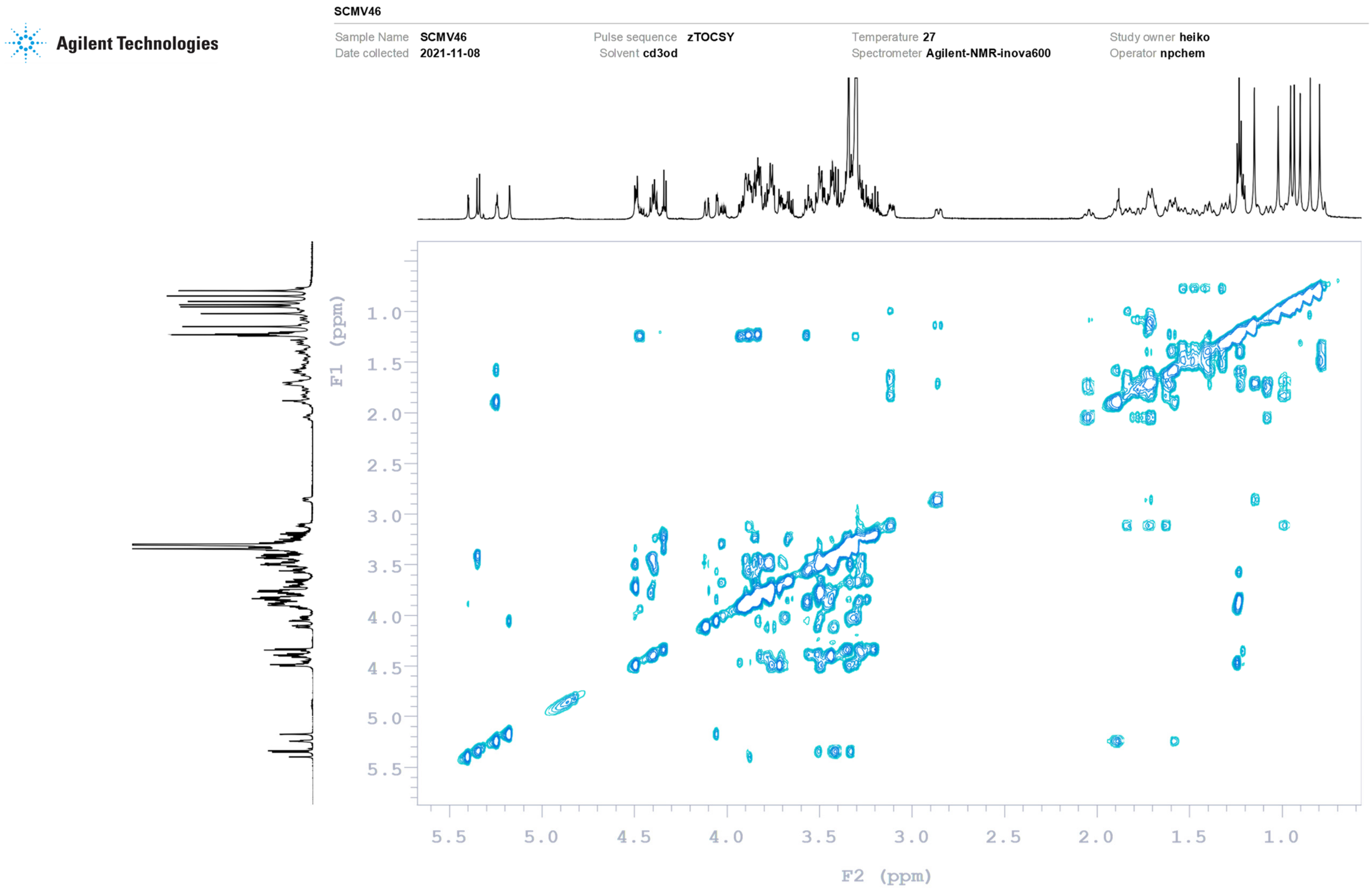

Figure S25 – TOCSY spectra of compound 5
